# A view-based decision mechanism for rewards in the primate amygdala

**DOI:** 10.1016/j.neuron.2023.08.024

**Published:** 2023-12-06

**Authors:** Fabian Grabenhorst, Adrián Ponce-Alvarez, Alexandra Battaglia-Mayer, Gustavo Deco, Wolfram Schultz

**Affiliations:** 1 Department of Experimental Psychology, University of Oxford, Mansfield Road, Oxford OX1 3TA, UK; 2 Department of Physiology, Development and Neuroscience, University of Cambridge, Downing Street, Cambridge CB2 3DY, UK; 3 Center for Brain and Cognition, Department of Technology and Information, Universitat Pompeu Fabra, Carrer Ramón Trias Fargas, 25-27, 08005 Barcelona, Spain; 4 Departament de Matemàtiques, EPSEB, Universitat Politècnica de Catalunya, Barcelona, 08028 Barcelona, Spain; 5 Department of Physiology and Pharmacology, Sapienza University of Rome, 00185 Rome, Italy; 6 Institució Catalana de la Recerca i Estudis Avançats, Universitat Barcelona, Passeig Lluís Companys 23, 08010 Barcelona, Spain

**Keywords:** decision making, choice, neural code, abstract representation, attractor, expansion recoding

## Abstract

Primates make decisions visually by shifting their view from one object to the next, comparing values between objects, and choosing the best reward, even before acting. Here, we show that when monkeys make value-guided choices, amygdala neurons encode their decisions in an abstract, purely internal representation defined by the monkey’s current view but not by specific object or reward properties. Across amygdala subdivisions, recorded activity patterns evolved gradually from an object-specific value code to a transient, object-independent code in which currently viewed and last-viewed objects competed to reflect the emerging view-based choice. Using neural-network modeling, we identified a sequence of computations by which amygdala neurons implemented view-based decision making and eventually recovered the chosen object’s identity when the monkeys acted on their choice. These findings reveal a neural mechanism in the amygdala that derives object choices from abstract, view-based computations, suggesting an efficient solution for decision problems with many objects.

## Highlights

• Different amygdala neurons code object-specific value or generalized view-based value
• Amygdala neurons code abstract view-based choice before coding object-specific choice
• A neural-network model derives object choices from abstract view-based computations
• View-based decision computations efficiently solve “many-objects” choice problems

## Graphical abstract

**Figure:**
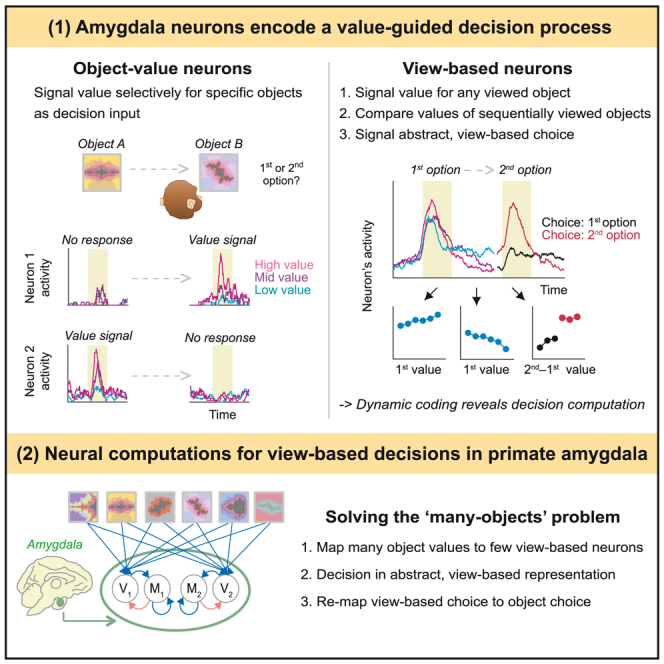

## Teaser

The amygdala is important for assigning value to objects. Grabenhorst et al. find that primate amygdala neurons translate object values into behavioral choices by implementing a decision mechanism. The amygdala’s decision mechanism uses abstract representations defined by the monkey’s current view rather than by specific object or reward properties.

## Introduction

To obtain rewards, primates make decisions visually. By shifting their view from one object to the next, they assess each object’s value, compare values between sequentially viewed objects, and decide on the best option from a distance, even before acting. A large body of evidence implicates the amygdala, a cell complex in the medial temporal lobe, in the valuation of visual objects.^1^^,^^2^^,^^3^^,^^4^ Yet, the amygdala’s role in translating object valuations into behavioral choices is poorly understood.

**Figure 1:**
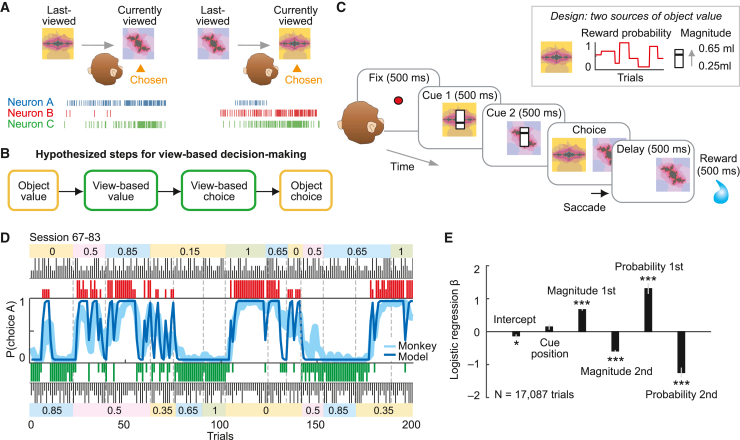
Conceptual framework for view-based decisions, choice task, and behavior (A) View-based decisions involve comparing currently viewed and last-viewed objects. Conventional decision neurons signal choices for specific objects (neurons A and B; ticks illustrate action potentials). View-based neuron C responds when the currently viewed object is chosen, irrespective of object identity (but would not respond when the last-viewed object is chosen). (B) Hypothesized information processing for view-based decisions. (C) Task: monkeys choose between sequentially viewed options based on reward values. Inset: object value derives from slowly changing, uncued reward probabilities and trial-specific, transiently cued magnitudes. 500-ms intervals separated both cues and choice period. (D) Example session. Trial-by-trial record of choices and rewards (red/green bars), running average of monkey’s choices, and choice probability of the reinforcement-learning model. Long/short colored bars, rewarded/unrewarded choices for objects A (red) and B (green); black bars, trial-specific magnitudes for object A (top) and object B (bottom); colored boxes, block-wise reward probabilities for objects A (top) and B (bottom). (E) Logistic regression (Equation 4) of choices for the first-viewed object in animal A (^∗∗∗^p < 0.001; ^∗^p < 0.05). See also Figure S1.

Here, we investigate the activity of primate amygdala neurons when monkeys make value-guided decisions between sequentially viewed objects. Decision computations are thought to involve winner-take-all competition between neurons encoding choices for specific objects, mediated by recurrent, mutual-inhibitory circuits.^5^^,^^6^^,^^7^^,^^8^ After the competition is resolved, decision neurons exhibit categorical, “on-off” activity patterns to signal whether a specific object is chosen (Figure 1A, neurons A and B). However, primate view-based decisions pose computational challenges for this scheme. Because primates evaluate objects sequentially through successive fixations,^9^ decision making requires a mechanism for comparing temporally separated value inputs. Further, this mechanism must process varying choice options flexibly to account for the vast number of objects that primates encounter. Although the primate brain stores large numbers of visual objects and their values,^10^^,^^11^^,^^12^ implementing competition for all possible object pairs would require myriad replications of object-specific decision circuits or highly flexible rescaling to new option sets.^6^^,^^8^

To avoid combinatorial explosion and preserve wiring economy,^13^ one solution might be to compute decisions not with object-specific neurons but with generalized, “view-based neurons” that can signal choice for any object that is currently viewed and attended to, irrespective of object identity (Figure 1A, neuron C). Different from object-specific neurons, which respond only to particular objects, view-based neurons would process choice in reference to the animal’s current view, or focus of attention, by responding flexibly to any currently viewed object. From this perspective, decision making would involve value-based competition between currently viewed and last-viewed objects,^14^^,^^15^ encoded as abstract representations independent of specific object features. Despite advances in understanding neural decision processes,^14^^,^^16^^,^^17^^,^^18^^,^^19^^,^^20^^,^^21^^,^^22^^,^^23^^,^^24^^,^^25^^,^^26^ it is unclear whether the primate brain contains a view-based decision mechanism for rewards. It is also unknown how neural systems could transform object values to view-based representations and subsequently recover the chosen object’s identity, which is critical for guiding behavioral choices (Figure 1B).

Neural encoding of view-based decisions as defined above implies an abstract representation of choice that is independent of specific properties of objects and rewards. Such abstract representations confer computational advantages, including generalization, emerge naturally in artificial neural networks trained on different tasks^27^ and exist in different cortical areas.^28^^,^^29^ However, it remains unclear whether abstract representations also underlie value-guided decisions and whether they exist in subcortical structures such as the amygdala.

We reasoned that the amygdala might be a suitable candidate area for implementing value-guided decision processes using abstract, view-based representations. The primate amygdala receives highly processed, object-level visual inputs^30^ and flexibly associates them with values.^1^^,^^2^^,^^31^ Consistently, amygdala lesions alter viewing preferences and reward-guided behaviors.^32^^,^^33^^,^^34^ Recent studies linked activity patterns in the rodent amygdala to specific actions and behavioral states.^35^^,^^36^^,^^37^^,^^38^ In primates, amygdala neurons have been directly implicated in decision making.^39^^,^^40^^,^^41^^,^^42^^,^^43^ Understanding the decision mechanism by which amygdala neurons link object valuations to behavior would have important implications, given the amygdala’s role in mental health disorders.^44^^,^^45^^,^^46^^,^^47^

## Results

### Monkeys make view-based decisions for rewards

We devised a task in which monkeys chose between sequentially viewed objects that differed in reward value (Figure 1C). This view-based decision task allowed us to test whether amygdala neurons encoded values and choices in a view-based representation, different from object-based representations, which might confer advantages for neural decision computations (Figures 1A and 1B). Importantly, the animals could form a choice for the currently viewed or last-viewed object covertly, before reporting it with a saccade. We initially tested neurons in the simplest scenario, involving two choice objects per session (A and B, presented in random viewing order), and later expanded object and reward sets. Optimal performance required integrating two value sources: tracking slowly varying object-reward probabilities from past experience and combining them with explicitly cued trial-specific magnitudes (Figure 1C, inset). To encourage view-based decisions, we cued magnitudes transiently during sequential viewing. (In additional tasks reported below, value derived only from reward probability or from varied reward types and magnitudes.) We used different colored fractals and natural images in each session as choice objects to engage amygdala neurons^48^ and distinguish view-based from object-based neuronal representations.

The monkeys successfully tracked object-reward probabilities and combined them with magnitudes when making their choices (Figures 1D and 1E). Mixed-effect logistic regression confirmed that choices depended on probabilities and magnitudes of both first- and second-viewed objects (Figures 1E; Equations 1, 2, 3, and 4). A reinforcement-learning model recovered the reward-probability estimates that guided the monkeys’ choices and confirmed that the monkeys approximated optimal learning (Figure S1; Tables S1 and S2). Object-value estimates derived from these models (Equation 4) integrated reward probabilities and magnitudes and were used as regressors for the neuronal analyses described below. The mean percentage of “correct” trials (without fixation breaks or other errors) was 64% ± 1% (animal A, 108 sessions) and 78% ± 1% (animal B, 36 sessions). Thus, the monkeys made reward-maximizing choices between sequentially viewed objects by comparing their values.

### Different amygdala neurons signal object value and view-based value

**Figure 2:**
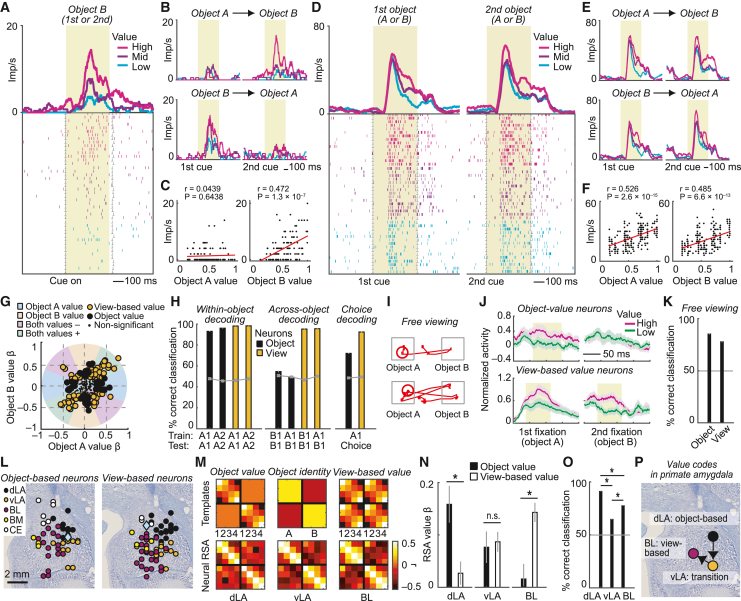
Distinct amygdala neurons encode object values and view-based values (A) Amygdala neuron encoding value for a specific object. Peri-event time histogram sorted by value terciles. Raster display: ticks indicate impulses, and rows indicate trials. Yellow area, analysis period. (B) The neuron encoded value for object B but not object A, irrespective of viewing sequence. (C) Linear regression of a neuron’s object-specific response on object values. (D) A different neuron encoding view-based value, irrespective of object identity. Responses to the first and second objects were sorted separately according to the values of the first and second objects. (E) The neuron encoded value for objects A and B, irrespective of viewing sequence. (F) Regression of neuron’s response on value. (G) Categorizing neurons as object-value coding (black) or view-based value coding (orange) from the angle in the space of value-regression coefficients (N = 233; analyzing first cue period). (H) Population decoding of value from neurons encoding object value (black bars, N = 31 neurons encoding object A value but not object B value) or view-based value (orange bars, N = 45 neurons) by training and testing the decoder on specific trial types (A1/A2: object A as first/second cue; B1/B2: object B as first/second cue; Choice: choice for currently viewed vs. last-viewed cue). Gray lines, shuffled data. (I) Measured eye positions during the saccade-choice period (“free viewing”) in two example trials. (J) Free-viewing activity of object-value neurons (top) and view-based neurons (bottom) during successive fixations (mean ± SEM). Yellow areas, p < 0.005, t test. (K) Population decoding of object value (averaged across objects) and view-based value (N = 233 neurons). (L) Histologically reconstructed recording sites for value-coding neurons. dLA, dorsal lateral nucleus; vLA, ventral lateral nucleus; BL, basolateral nucleus; BM, basomedial nucleus; CE, central nucleus. (M) RSA. Top: templates define similarity patterns for each variable; numbers 1 to 4, low to high value levels (quartiles); A/B, responses to objects A/B. Bottom: neural RSA matrices for all neurons in dLA, vLA, and BL. Colors indicate correlation coefficient for condition pairs, calculated between population-activity vectors. Condition-order was preserved across matrices. (N) Multiple regression of neuronal RSA on templates (^∗^p < 0.005). (O) NN value decoding (mean ± SEM; ^∗^p < 0.001, Wilcoxon test; N = 20 neurons per nucleus; analyzing first-cue period). (P) Summary schematic. See also Figures S2–S4.

We identified two types of value-coding neurons in amygdala that seemed to play complementary roles in decision making. “Object-value neurons” encoded value selectively for specific visual objects: a neuron would respond to its “preferred” (i.e., encoded) object—but not the alternative object—with a graded signal that depended on the object’s current value, irrespective of viewing sequence (Figures 2A–2C). By contrast, “view-based neurons” were not object selective, as they responded to both sequentially viewed objects by signaling the value of whichever object was currently viewed (Figures 2D–2F). We used a multiple-regression approach based on the angle of value regression coefficients (Figure 2G) to identify neurons that encoded object values (48 of 233 recorded neurons, 21%) and view-based values (69 neurons, 30%, p < 0.05, t test on regression coefficients, Equations 5 and 6; Figure S2) at the first choice cue. Value coefficients varied along a continuum (Figure 2G) but allowed for clear separation of object-value and view-based value neurons (Figure S2). Identification of both types of value-coding neurons was robust across analysis approaches (53/80 object-value/view-based value neurons identified at second choice cue; 42/71 neurons when collapsing cue periods; 54/97 neurons when estimating coefficients for both objects in one model; 45/64 neurons when including a chosen-value covariate).

Amygdala population activity encoded both types of value with high accuracy, quantified by a support-vector-machine (SVM) classifier (Figure 2H, left; cross-validated performance; p < 0.001, Wilcoxon test compared to shuffled data). However, only view-based neurons enabled flexible value decoding for different objects: a classifier trained on a particular object correctly decoded the alternative object’s value only with data from view-based neurons but not from object-value neurons (Figure 2H, middle; decoding with preselected object- or view-based neurons; see Figure S2 for results with unselected neurons). Moreover, only view-based neurons enabled accurate prediction of the monkeys’ choices, whereas choice-prediction based on object-specific neurons was much less accurate (Figure 2H, right; Figure S2). Thus, although object-value neurons signaled object-specific values as important decision inputs, they could not directly encode value comparisons because they did not process the value of the alternative object. By contrast, view-based neurons processed values for both objects, implicating them in value comparisons and decision making.

We confirmed the presence of object- and view-dependent value signals by examining neuronal activity during the saccade-choice period. In this period, both objects appeared simultaneously, and the animals freely looked back and forth between them before indicating their choice, evidenced by measured eye positions (Figure 2I). During consecutive fixations, both object-specific and view-based value signals re-emerged: object-value neurons signaled value only when the animal fixated the neuron’s encoded object but not when fixating the alternative (Figure 2J, top). By contrast, view-based neurons signaled the value of whichever object the monkey currently fixated (Figure 2J, bottom). Consistently, population activity during free viewing allowed for accurate decoding of object values and view-based values (Figure 2K). Thus, activity recorded during passive and active sequential viewing showed that amygdala neurons signaled object values and view-based values.

### A transition between value codes across amygdala nuclei

Although value neurons were prevalent in different amygdala subdivisions (Figures 2L; and S3), we found evidence for a topological transition from object- to view-based value codes that followed the amygdala’s internal connectivity.^49^ We identified this “object-to-view transition” using representational similarity analysis (RSA), which quantifies the similarity (i.e., correlation) of neuronal responses between different conditions (e.g., specific objects, value levels) to characterize population codes.^17^^,^^50^

RSA revealed a primarily object-specific value code in the dorsal part of the lateral nucleus (dLA), the amygdala’s sensory entry point and storage site for stimulus-value associations.^30^^,^^46^ Specifically, dLA activity discriminated values for specific objects (on-diagonal block structure of the RSA matrix), but responses to different objects were unrelated (lack of off-diagonal block structure), indicating object-value coding (Figures 2M, 2N, left, and S4; regressors included object identity, object value, view-based value; significance based on permutation tests, see STAR Methods). By contrast, in the basolateral nucleus (BL), a downstream structure with distinct inputs,^30^ responses to different objects were related, indicating view-based coding according to RSA. Specifically, the BL population represented values of sequentially viewed objects as anti-correlated activity patterns (off-diagonal matrix structure; Figures 2M and 2N, right), indicative of value comparisons. The ventral lateral nucleus (vLA), an intermediate structure, showed a mixture of object- and view-based codes (Figures 2M and 2N, middle). Additional analyses confirmed the robustness of nucleus-specific findings (Figure S4). A biologically plausible nearest-neighbor (NN) decoder revealed that the object-value code in dLA was particularly accurate because it used well-separated activity patterns to represent different value levels for specific objects (Figures 2O and S4).

Thus, value codes gradually transitioned across amygdala subdivisions: an object-based code in dLA accurately tracked values for specific objects and transitioned to a view-based code in BL that could support decision making through value comparisons (Figure 2P: identified value codes in different nuclei; arrows indicate hypothesized information flow, requiring confirmation in future studies). We next investigated how amygdala neurons processed these values into choices.

### An abstract, view-based choice signal in amygdala neurons

**Figure 3:**
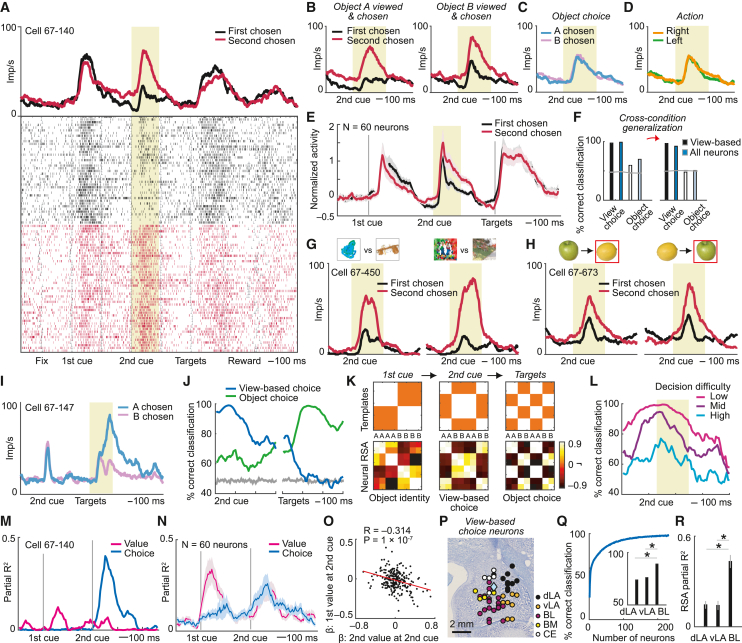
A view-based decision process in amygdala neurons (A) Amygdala neuron encoding view-based choice (i.e., choice for currently viewed vs. last-viewed object) during second cue. (B–D) Activity of the same neuron sorted by object choice and viewing sequence (B), object choice (C), and action (saccade direction) (D). (E) Population activity of view-based choice neurons (selected by sliding-window regression; mean ± SEM). (F) Population choice decoding during second cue. Left: decoding view-based choice and object choice from all trials; right: cross-condition decoding using different object-viewing sequences for decoder training and testing. Black/white, 39 pre-selected neurons encoding view-based choice during second cue; blue/pale blue, 233 unselected neurons. (G) Neuron encoding view-based choice in control experiment with four different visual objects. (H) Neuron encoding view-based choice in control experiment with two different rewards (preferred apple vs. non-preferred lemon juice). (I) Neuron encoding object choice during presentation of saccade targets. (J) Population decoding of view-based choice (blue) and object choice (green) from all neurons (N = 233; mean ± SEM). Gray lines, shuffled data. (K) RSA for object identity, view-based choice, object choice during first cue, second cue, targets. Top: templates (letters A/B in left panel indicate object identity; letters A/B in middle and right panels indicate object choice); bottom: neuronal RSA matrices (N = 233 neurons). (L) Choice decoding for value-difference terciles (N = 60 view-based choice neurons; mean ± SEM; p < 0.001, Wilcoxon test). (M) Value-to-choice transition in the neuron shown in (A). (N) Value-to-choice transitions in view-based choice neurons (mean ± SEM). (O) Anti-correlated neuronal value coefficients between first and second cues indicate value comparison (N = 233). (P) Recording sites of view-based choice neurons. (Q) Decoding view-based choice during second-cue period for different decoding sample sizes (mean ± SEM). Inset: nucleus-specific decoding (N = 20 neurons per nucleus; ^∗^p < 0.001, Wilcoxon test). (R) Nucleus-specific RSA regression of view-based choice during second cue (^∗^p < 0.005). See also Figures S5–S9.

To instruct actions, choices must ultimately refer to specific objects. Accordingly, neurons should encode the decision outcome by signaling the chosen object’s identity. Many amygdala neurons encoded such conventional object-choice signals, consistent with previous reports.^39^^,^^41^^,^^42^^,^^43^ However, we also observed earlier, transient choice signals that were not object based but that may constitute precursors to object-choice signals. These signals encoded the monkey’s choice in an abstract, purely “internal” activity space referenced to the monkey’s current view but not to external objects, rewards, or actions (Figure 3). View-based choice signals preceded object-choice signals, were insensitive to physical object and reward features, and carried signatures of decision computation, as described next.

When the monkey viewed the second of the two sequentially presented objects, the amygdala neuron in Figure 3A showed differential activity that depended on the view-based choice the monkey was going to make on a given trial. Specifically, the neuron was strongly active when the monkey chose the currently viewed, second object, irrespective of its identity, but had reduced activity when the monkey chose the previously viewed, first object. This view-based choice signal occurred equally on trials when the monkey chose object A (i.e., when A was the second object) or object B (when B was second; Figure 3B), confirming that the choice signal was view based but not linked to a particular object. Accordingly, the neuron signaled view-based choice for currently viewed objects without signaling object choice (Figure 3C) or forthcoming left-right action (Figure 3D). Multiple regression confirmed that the neuronal response was only explained by view-based choice (p = 2.0 × 10^−14^) but not by chosen object (p = 0.69), value (p = 0.15), action (p = 0.32), cued reward magnitude, or other variables (Equations 7 and 8). Among 233 amygdala neurons, 60 neurons (26%) encoded view-based choice with such activity patterns (Figures 3E; Equation 7; 54 neurons when controlling for reward magnitude; Equation 8). Of these, 33 neurons showed higher activity when the second option was chosen, and 27 neurons showed higher activity when the first option was chosen (p = 0.438, z test). View-based choice signals occurred even on trials in which the first- and second-viewed options were matched for reward magnitude or value (Figure S5).

Amygdala neurons encoded view-based choice during the second-cue period with high accuracy (97% and 99% correct cross-validated SVM classification for 39 pre-selected neurons and 233 unselected neurons, respectively), whereas object-choice encoding in this early trial epoch was near chance (Figure 3F, left). We investigated whether the view-based choice code generalized across object-viewing sequences by testing the cross-condition generalization performance, a criterion for an abstract, stimulus-independent representation.^29^ We trained the classifier to decode view-based choice from one object-viewing sequence (e.g., A then B) and tested performance on the alternative sequence (B then A) not provided to the classifier during training. Consistent with an abstract representation, classification for view-based choice (but not object choice) generalized across viewing sequences (Figure 3F, right).

The view-based choice signals described here are remarkable because they could reflect an efficient decision mechanism that processes abstract choice representations irrespective of object features. Therefore, we next tested the independence of view-based choice signals from physical object and reward properties.

### View-based choice signals are independent of physical object and reward properties

In two control experiments, we varied the physical features of both visual and reward objects. First, we tested neurons with two sets of visual choice objects (see STAR Methods; value derived only from object-specific reward probabilities). Individual amygdala neurons encoded view-based choice across object sets; i.e., a given neuron signaled whether the monkey would choose the currently viewed object, irrespective of which of four visual objects was chosen (Figure 3G; 34/205 neurons, 17%; Equation 10). Second, we introduced different physical rewards that elicited subjective preferences (e.g., preferred apple juice vs. non-preferred lemon juice), tested under changing cue-reward associations (Figure S6; see STAR Methods; value derived from reward type and reward magnitude). Individual amygdala neurons signaled view-based choice irrespective of which specific reward was chosen or which visual cue indicated the reward (Figure 3H; 17/72 neurons, 24%; Equation 11). In all three tasks, view-based choice signals fulfilled the criterion of an abstract representation^29^: choice-decoding performance generalized across conditions, even when classifiers were trained and tested on different object sets or physically different rewards (Figure S7). Thus, experimental tests confirmed that view-based choice signals in amygdala neurons were independent of visual object identity and physical reward characteristics.

### View-based choice signals precede object-choice signals

We also observed conventional object-choice signals; however, these typically followed view-based choice signals. When the saccade targets appeared, the neuron in Figure 3I signaled whether the monkey would choose object B over object A (p = 1.0 × 10^−16^, Equation 7); it did not carry an earlier, view-based choice signal (p = 0.53). Of 233 neurons, 94 neurons (40%) explicitly encoded object choice, often without encoding view-based choice (57/94 neurons). Population decoding showed a clear transition from early view-based choice coding, which peaked when the monkey viewed the second object, to subsequent object-choice coding when the choice targets appeared (Figure 3J; value and choice neurons contributed to decoding). This transition was also evident in the population representational similarity structure: population activity initially reflected the first object’s identity (Figure 3K, left) before evolving into a transient, view-based choice code during the second cue (Figure 3K, middle) and then transitioning to an object-based choice code during target presentation (Figure 3K, right). These effects were present in time-resolved RSA patterns and robust across two animals and three experimental tasks (Figure S8). Object-choice signals provided a useful control that our task did not pre-determine view-based choice signals. Thus, transient view-based choice signals preceded object-choice signals, suggesting that decisions were initially computed in a view-based representation.

### Amygdala neurons encode a view-based decision computation

View-based choice signals in amygdala reflected the critical, well-conceptualized signatures of a decision computation.^5^^,^^6^ First, in formal decision models, the strength of the choice signal—the decision output—increases for easier decisions due to a clearly resolved winner-take-all competition.^5^ We confirmed this pattern in view-based choice signals: neuronal discrimination of the animal’s view-based choice increased with decreasing decision difficulty, which is inversely related to the value difference between options (Figure 3L; both view-based value and choice neurons contributed to this decoding).

Second, decision neurons should reflect transitions from coding the evidence entering the decision process (i.e., the values) to coding the binary choice.^5^^,^^51^ Following this principle, the binary view-based choice signal of the neuron in Figure 3A was preceded by a graded value signal, indicated by an early peak in the time-resolved partial-regression coefficient for value (Figures 3M; Equation 7). Such value-to-choice transitions occurred in 34 of 60 view-based choice neurons (56.6%; Figure 3N) and in control experiments extended to multiple visual and reward objects (Figure S9). By translating value inputs to choice outputs, the activity patterns of these neurons matched the information flow of computational decision models.^5^

Third, amygdala neurons directly encoded value comparisons between currently viewed and last-viewed objects. Neuronal value coefficients were anti-correlated between first- and second-viewed objects, indicating that competing choice options had opposing influences on neuronal activity (Figures 3O and S9; Equation 9, controlled for value range and intrinsic value anti-correlation; Figure S9). By contrast, neurons did not reflect value comparisons based on object identity (Figure S9). Taken together, these activity patterns were consistent with an underlying view-based decision computation in amygdala neurons.

Although we found view-based choice signals throughout amygdala nuclei (Figure 3P), they were strongest in BL. Choice-decoding accuracy generally increased as more neurons entered into the decoder (Figure 3Q), but BL neurons were the most precise in discriminating view-based choices (Figure 3Q, inset). Signatures of value comparison and cross-condition generalization of choice decoding were also strongest in BL (Figure S9). Importantly, the view-based choice code in BL was particularly distinct, accounting for up to 43% of explained variance in representational similarity structure compared to 15% in the lateral nucleus (LA) (Figures 3R and S9). These results identify BL as a key amygdala site for view-based decision computation.

### A three-stage neural mechanism for view-based decisions

**Figure 4:**
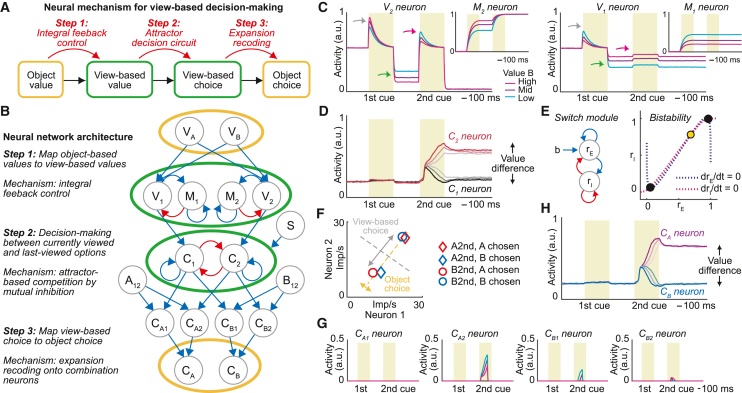
Neural-network model for abstract, view-based decisions in amygdala (A) Computations for view-based decision making informed by recorded amygdala neurons. (B) Architecture of the biologically plausible model implementing view-based decision making. Circles indicate pools of functional neuron types. Blue/red, excitatory/inhibitory connections. (C) Model neurons encoding view-based value with positive (left) and negative (right) tuning. Inset: memory neurons implementing integral feedback control. In these simulated trials, the first value was higher than the second, constant value. (D) Model neurons encoding view-based choice across difficulties (i.e., value difference). (E) Switch mechanism initiates decision computation. Attractor dynamics of view-based decision neurons depend on excitatory drive from switch neurons (“S” in B). Left: switch module with interconnected excitatory and inhibitory populations. Right: the system’s low-activity and high-activity branches coexist in a region of bistability. State transitions depend on applied input. In the plotted bistability region, the system has two stable points (black) and one unstable point (yellow). (F) The amygdala’s view-based code maximizes discriminability for view-based choice (gray arrow) but limits discriminability for object choice (orange arrow). Data from two typical view-based amygdala neurons. (G) Combination neurons signal object choice for specific object-viewing sequences, shown for trials in which object A was viewed second and chosen. (H) Model neurons encoding object choice. See also Figures S10–S13.

Our data are consistent with the notion that the primate amygdala encodes a view-based decision process (cf. Figure 1B). To explain this process mechanistically, we designed a biologically plausible neural-network model that combined three well-defined circuit computations to reproduce our recorded amygdala signals (Figures 4A, 4B, and S10). As described next, the model implements view-based decision making in three stages: (1) mapping object- to view-based values via integral feedback control; (2) computing abstract, view-based choice via winner-take-all competition through mutual inhibition and attractor dynamics; and (3) mapping view-based choice to object choice via expansion recoding.

The model’s first stage converts object-value inputs to view-based values and stores them in short-term memory for value comparison between sequential options. When viewing the first object (A or B), object-value neurons (V_A_ or V_B_) activate two pools of oppositely tuned view-based value neurons (Figure 4B, V_1_, negative value tuning; V_2_, positive value tuning) that encode the first object’s value irrespective of object identity (Figure 4C, gray arrows). Importantly, decision making between sequentially viewed objects requires memory for the first object’s value. For this purpose, view-based value neurons interact with recurrent “memory neurons” (M_1_, M_2_, Figures 4B and 4C, insets) that provide sustained inhibitory feedback proportional to the first object’s value (“integral feedback control”^52^). Thus, after viewing the first object, the M-to-V circuits maintain the object’s value throughout the inter-stimulus interval, with higher (V_1_) or lower (V_2_) activity for larger values, respectively (Figure 4C, green arrows). When viewing the second object, the sustained M-to-V inhibition modulates the response of the positively tuned V_2_ neurons, producing strong responses if the value input by the second object overcomes the inhibition proportional to the first object’s value (Figure 4C, magenta arrows). Conversely, the negatively tuned V_1_ neurons respond strongly only if the second value input is smaller than the first. This gating of the second-object response by sustained inhibition allows view-based neurons to provide comparison evidence for the two temporally separated values to downstream decision neurons ( Figure S10). Thus, the first model stage uses integral feedback control to transform object values to view-based values and prepare them as decision inputs.

The second model stage performs the view-based decision computation. Two distinct pools of decision neurons process value input from the preceding stage’s V_1_ and V_2_ neurons (Figure 4C), predisposing them to encode choice for currently viewed objects (C_2_) and last-viewed objects (C_1_), respectively (Figure 4D). Based on the strength of the value inputs, the neurons compete with each other via a winner-take-all process implemented through mutual inhibition and recurrent excitation.^5^ This decision circuit amplifies differences between conflicting value inputs until the “winning” neuronal pool enters a stable attractor state that signals view-based choice and suppresses the alternative pool. Similar to previous models,^5^^,^^53^ attractor dynamics produce choice signals that reflect decision difficulty, with stronger signals resulting from clearly resolved competitions (Figure 4D). Different from previous models, decision neurons in our model do not encode choices for specific objects but instead compute choices in an abstract, purely internal activity space, referenced to the animal’s view rather than to sensory objects or planned actions. To prevent premature decision computation, competition is only initiated once the second object is viewed, mediated by excitatory drive from bistable “switch neurons” (S) that integrate consecutive object inputs (Figures 4B, 4E, and S11). Thus, the second model stage computes object-independent, view-based choices through winner-take-all competition by recurrence and mutual inhibition.

Choice signals ultimately serve to direct actions toward chosen objects. However, view-based choice signals are referenced to neither objects nor actions but instead mix information about the chosen object and the object-viewing sequence. Importantly, the view-based code provided by amygdala view-based choice neurons is too compressed to enable a downstream neuron (or other linear decoder) to read out the chosen object’s identity directly (Figure 4F, cf. Figure 3F). Accordingly, view-based choice signals must be mapped back to an explicit object-based representation. For this purpose, in the final model stage, view-based choice signals are projected onto an expanded space of intermediate “combination neurons” that help solve the mapping from view-based to object-based choice signals (Figures 4B and 4G). These neurons (C_A1_, C_A2_, C_B1_, C_B2_) combine inputs about view-based choice from the second stage with separate inputs from object-sequence neurons (A_12_, B_12_) that signal the sequence in which objects A and B had been viewed. We found object-sequence signals in recorded amygdala neurons (Figure S12) and modeled them using synaptic depression (see STAR Methods). Combination neurons thus serve as “expansion-recoding devices”^54^ that recover the necessary object information for signaling view-based choice for specific objects. In a final step, these signals converge onto conventional object-choice neurons (C_A_, C_B_) that explicitly signal the choice for object A or object B, irrespective of viewing sequence (Figures 4B and 4H).

### Amygdala neurons carry signatures of model computations

**Figure 5:**
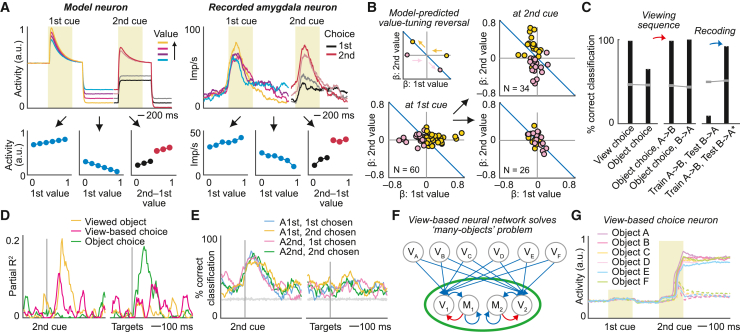
Amygdala neurons encode signatures of model computations (A) Model neuron and recorded amygdala neuron from Figure 3A reverse value tuning from the first cue to delay, indicating integral feedback control. At the second cue, neurons encode the chosen option more strongly for easy (saturated colors) than difficult (faint colors) decisions. (B) Value-tuning reversal from first to second cue in recorded view-based choice neurons (N = 60). Inset: colored arrows indicate model-predicted value-coefficient changes. Top right: neurons encoding value-to-choice conversions. Bottom right: neurons encoding view-based choice but not value. (C) View-based amygdala neurons (N = 60) enable accurate decoding of view-based choice, but not object choice, during second cue (left). Object choice can be decoded for specific object-viewing sequences (middle), but not across viewing sequences (“train A→B, test B→A”). Recoding classification input by viewing sequence recovers object-choice decoding (right, “train A→B, test B→A^∗^”). (D) Amygdala neuron encoding model-predicted conjunction. (E) Decoding model-predicted conjunctions (N = 233 neurons; mean ± SEM). Gray lines, shuffled data. (F) Model extension to decision making between many objects. (G) Model neuron encodes view-based choice for many objects. Solid/dotted lines, object chosen/not chosen. See also Figure S14.

Our model’s computations predicted specific activity patterns that were confirmed by experimental data. One key prediction derives from the operation of integral feedback control^52^ and attractor-based competition.^5^^,^^6^ View-based neurons with positive value tuning during the first cue (V_2_) should reverse their value tuning during the delay period, because of sustained value-dependent inhibition by M neurons, and evolve into a choice signal whose strength reflects decision difficulty (Figure 5A, left), consistent with winner-take-all competitive selection. Remarkably, the view-based amygdala neuron from Figure 3A showed precisely this pattern (Figure 5A, right), supporting the operation of integral feedback control and attractor-based competition in amygdala. The encoding of decision difficulty (Figure 3L), the timing of value-to-choice transitions (Figures 3M and 3N), and anti-correlated value coefficients between first and second options (Figure 3O) confirmed these predictions across amygdala neurons.

Importantly, the dynamic coding pattern by which neuronal value tuning reversed from the first to the second option (Figure 5B) was consistent with integral feedback control,^52^ but not with alternative mechanisms,^53^^,^^55^ with 80% of view-based neurons (48/60 neurons) showing this consistent pattern (Z = 4.65, p = 3.3 × 10^−6^). Notably, value-tuning reversal was more pronounced in neurons encoding value-to-choice transitions (Figure 5B, top right) compared to pure view-based choice neurons (Figure 5B, bottom right) and was evident in population activity (Figure S10), in support of separate pools of view-based value and choice neurons in our model (Figure 4B). Amygdala value signals were phasic but varied in latency and duration, thereby tiling the delay period between sequential objects and enabling accurate value decoding (Figure S10). Consistently, modeled activation levels of M neurons could vary while retaining the network’s ability to compare sequential stimuli (Figure S13).

Neuronal data confirmed the necessity and feasibility of a recoding step to read out object choice from the view-based code: across all trials, the amygdala’s view-based code enabled accurate readout only for view-based choice, but not for object choice (Figure 5C, left), indicating that neural activity was separated along a view-based choice axis but compressed along the object-choice axis (cf. Figure 4F). Object choice could be read accurately when holding the object-viewing sequence constant (Figure 5C, middle); however, when training and testing the decoder on alternative viewing sequences, object choice was systematically misclassified (Figure 5C, right). Object-choice information could be recovered when providing the decoder with information about viewing sequence (Figure 5C, right), by recoding classification labels according to viewing sequence (see STAR Methods). Evidence for recoding was strongest in BL compared to dLA and vLA (Figure S14). Our model suggests that this recoding step involves expansion recoding onto combination neurons, supported as follows. Amygdala neurons encoded the model-predicted three-way conjunctions of viewing sequence, view-based choice, and object choice (Figure 5D). Specifically, of 60 neurons encoding view-based choice, 37 and 27 neurons also encoded viewing sequence and object choice, respectively. A multi-class SVM decoder accurately read these conjunctions from population activity (Figure 5E).

In summary, the neural-network model uses integral feedback control to map object-specific values onto abstract, view-based representations that implement a competitive decision process. After view-based choice is computed, the chosen object’s identity is recovered via expansion recoding. Activity patterns of recorded amygdala neurons, including model-predicted tuning dynamics, choice codes, and signal conjunctions, supported the model’s key computations.

## Discussion

Primates make decisions visually by shifting their view from one object to the next to compare the objects’ values and form an internal choice before acting. Our data suggest that amygdala neurons support view-based decision making by deriving abstract, view-based choices from object-specific values via a sequence of well-defined neural-circuit computations. These findings establish a role for the primate amygdala in decision making that extends considerably beyond object valuation.

Different amygdala neurons signaled value selectively for specific objects or unselectively for whichever object the monkey currently looked at. By commonly referencing values of different objects to the monkey’s view, these latter view-based neurons enabled an abstract decision computation that compared currently viewed and last-viewed objects irrespective of their identity. Individual neurons encoded this decision computation through (1) opposing, dynamic value tuning for sequentially viewed objects, consistent with integral feedback control^52^; (2) signatures of winner-take-all competition^5^^,^^6^; and (3) value-to-choice transitions, directly linking decision inputs and outputs. This object-to-view transformation was computationally efficient: it generalized across task contexts and allowed the same neurons to signal choices for different objects, rewards, and actions.

View-based choice signals in amygdala differ markedly from known object-choice signals^39^^,^^41^^,^^42^^,^^43^: they predicted whether the monkey would choose the currently viewed object irrespective of object identity and forthcoming action, preceded conventional object-choice signals, and were shown in control experiments to be independent of physical object and reward features. These properties also differ from action-specific choice signals in sequential vibrotactile decision tasks.^51^ Notably, our task could, in principle, be solved using a purely object-based code; indeed, some neurons encoded values and choices for specific objects. Thus, our finding that amygdala neurons encoded values and choices independently of object features, before encoding object choice, was not pre-determined by task design. Moreover, view-based choice signals were found across tasks with varying features, such as whether value derived from reward probability, magnitude, or type. Arousal or attention did not explain view-based choice signals as neurons showed computationally well-defined value-to-choice transitions and predicted choice on value-matched trials. In being detached from sensorimotor contingencies and generalizing across task conditions (Figures 3F–3H), view-based choice signals fulfilled criteria for an abstract, cognitive representation^29^^,^^56^ that could serve as an interface between valuation and action. Consistent with this notion, view-based choice signals were transient: shortly before the animals acted on their choice, neural activity reverted to an object-referenced code that identified the chosen object.

To guide actions, neurons should signal the chosen object unambiguously. Although amygdala neurons signaled view-based choice with high accuracy, this view-based code was too compressed for a linear decoder (e.g., a downstream neuron) to read out the chosen object’s identity. Recovering object information required a recoding step to increase the linear separability of neuronal object-choice patterns. Our model solved this problem by expansion recoding onto combination neurons that mixed information about view-based choice and object-viewing sequence. This mechanism is supported by recorded model-predicted combination neurons, mixed coding in amygdala,^3^^,^^35^ and the amygdala’s internal feedforward circuitry,^49^^,^^57^ which could generate such mixed representations.

The identified abstract, view-based decision mechanism in amygdala is computationally efficient. It solves the problem of comparing many objects with few decision neurons and thus avoids combinatorial explosion from replicating mutual-inhibitory circuits for all pairwise object comparisons. While alternative solutions to many-objects decision problems exist, including fine-tuned inhibition or rescaling to new option sets,^6^^,^^8^ the mechanism shown here is supported by our single-neuron data and consistent with known amygdala inhibitory, recurrent and feedforward connections.^49^^,^^57^^,^^58^ Although our model introduces complexities of mapping object to view-based values and remapping view-based to object choices, these operations can be implemented by common feedforward connectivity^49^^,^^57^ and competitive networks.^59^ Importantly, our model can be readily extended to process many objects (Figure 5F). Neuronal data (Figure 3G) and simulations (Figure 5G) show that the proposed mechanism flexibly processes values from multiple objects to compute view-based choice, provided full connectivity is specified in the first, object-to-view stage.

Previous attractor models implemented sequential decision making with flexible control of mutual inhibition,^53^ integral feedback control,^52^ or synaptic facilitation.^55^ Among these variants, only the mechanism by Miller and Wang,^52^ which we incorporated here, predicts value-tuning reversal between sequential options, as observed in our data. Different from attractor models, reservoir models that adjust network connectivity or readout also produce delay activity that is heterogeneous, dynamical, and reverses tuning.^60^^,^^61^ An advantage of reservoir computing is that it does not require precise connectivity tuning for integral feedback control or mutual inhibition. Future extensions of these models could incorporate the expansion from view-based to object-specific choice representations suggested here.

Our data indicate functional differences between primate amygdala nuclei that so far remained elusive.^3^ We found that dLA neurons carried a highly accurate, object-centric value code that transitioned, via an intermediate stage in vLA, to a predominantly view-based code in BL (Figures 2M–2P). dLA specialization for object valuation aligns with this structure’s rich visual inputs,^30^ highly selective object responses,^48^ and known function in associating stimuli with value.^45^^,^^46^ In our model, object values were converted to view-based values through feedforward convergence and integral feedback control (Figure 4B). This proposal is consistent with the predominantly feedforward projections from dLA to BL,^49^ prefrontal BL inputs^30^ that could contribute to integral feedback control,^52^ and proposals that BL neurons elaborate cue-evoked LA responses.^57^ Remarkably, BL neurons predicted view-based choices much more precisely than dLA and carried a stronger view-based choice code (Figures 3Q and 3R). Thus, valuation and decision processes may map onto partly distinct amygdala subdivisions, with BL acting as the primary site for decision computation. A caveat to these conclusions is that we did not examine the relative timing of information processing across nuclei with simultaneous recordings.

In previous studies, neural decision processes involved either object-centric or sequential reference frames.^14^^,^^17^^,^^18^^,^^19^^,^^62^ Our data help reconcile these observations. We show that object- and view-based signals serve complementary functions in decision making and coexist in a single brain structure and even in single neurons and that transitions between these reference frames emerge from specific circuit computations. Our finding that view-based choice signals preceded object-choice signals supports proposals that view-based decision variables are not simply a corollary of attention but play a central role in the computation of economic choice.^14^^,^^15^

Importantly, we do not suggest that the amygdala processes decisions independently of other brain systems or that primates exclusively use a view-based decision strategy. Decision making engages additional processes that involve other brain structures, including the prefrontal cortex,^9^^,^^16^^,^^17^^,^^18^^,^^19^^,^^20^^,^^21^^,^^22^^,^^23^^,^^24^^,^^63^^,^^64^ which interacts with the amygdala during decision making.^31^^,^^34^^,^^65^^,^^66^

A classical study identified primate amygdala neurons that tracked values of visual stimuli in a Pavlovian context, which included neurons that did not encode stimulus identity.^2^ Our results in a choice task suggest that such neurons are particularly important for comparing values between sequentially viewed objects. View-based value signals differ from previously reported option-specific and chosen-value signals^19^^,^^39^^,^^41^: although they reflected subjective, economic value as in previous studies, they did not reflect option identity or choice and were detected in regressions that controlled for these variables.

How does the identified decision mechanism contribute to amygdala function? As shown here, view-based representations generalize across task contexts, similar to other abstract representations.^28^^,^^29^ Accordingly, view-based choice signals could focus different output functions on currently viewed rewards, including amygdala-coordinated motivational states^35^^,^^37^^,^^38^ and primate social-gaze interactions.^4^^,^^47^^,^^67^ Our results predict that amygdala damage should disrupt view-based valuations, which may explain altered viewing preferences following amygdala lesions.^32^^,^^33^ In generalizing across objects, rewards, and contexts, the amygdala’s view-based mechanism also constitutes a vulnerability for dysfunction that could help explain generalized reward-valuation deficits in depression, in which the amygdala is implicated.^68^

In summary, our findings uncover a neural mechanism in the primate amygdala that derives object choices from abstract, view-based computations. By implementing value-guided competition between sequentially viewed options, this mechanism seems suited for primate-typical decision making through visual fixations.^9^^,^^14^^,^^17^^,^^20^ Our single-neuron data and neural-network model using abstract representations could inform the design of adaptive decision systems that efficiently solve many-objects choice problems, which challenge both biological and artificial intelligence.

## STAR★Methods

### Key resources table

**Table:** 

REAGENT or RESOURCE	SOURCE	IDENTIFIER
**Experimental models: Organisms/strains**		

Rhesus macaques (Macaca mulatta)	Centre for Macaques (CfM)	N/A

**Software and algorithms**		

MATLAB	MathWorks	http://mathworks.com
Plexon offline sorter	Plexon	http://plexon.com
Computational model	This paper	https://github.com/FGrabenhorst/Grabenhorst_AmygdalaChoice_Neuron2023/releases/tag/v1.0 (https://doi.org/10.5281/zenodo.8268856)

**Other**		

Microelectrodes	FHC	http://www.fh-co.com/
Double asymmetric head holder, Recording chamber, Low-profile bone Screws	Gray Matter Research	http://www.graymatter-research.com
NHP TV-front chair	Crist Instruments	http://www.cristinstrument.com
MO-90 manual micromanipulator electrode drive	Narishige	https://uk.narishige-group.com

### Resource availability

#### Lead contact

Further information and requests for reagents and resources should be directed to and will be fulfilled by the lead contact, Dr. Fabian Grabenhorst (fabian.grabenhorst@psy.ox.ac.uk).

#### Materials availability

This study did not generate new unique reagents.

### Experimental model and subject details

Two adult male rhesus monkeys (Macaca mulatta) weighing 10.5 and 12.3 kg participated in the present experiments. The number of animals used is typical for primate neurophysiology experiments. The animals were on a standard diet for laboratory macaques and had free access to the standard diet before and after the experiments. During the experiments, the animals received their main liquid intake in the laboratory. All animal procedures conformed to US National Institutes of Health Guidelines. The work has been regulated, ethically reviewed and supervised by the following UK and University of Cambridge (UCam) institutions and individuals: UK Home Office, implementing the Animals (Scientific Procedures) Act 1986, Amendment Regulations 2012, and represented by the local UK Home Office Inspector; UK Animals in Science Committee; UCam Animal Welfare and Ethical Review Body (AWERB); UK National Centre for Replacement, Refinement and Reduction of Animal Experiments (NC3Rs); UCam Biomedical Service (UBS) Certificate Holder; UCam Welfare Officer; UCam Governance and Strategy Committee; UCam Named Veterinary Surgeon (NVS); UCam Named Animal Care and Welfare Officer (NACWO).

### Method details

#### Neurophysiological recordings

We used experimental procedures for neurophysiological recordings from the amygdala in awake, behaving macaque monkeys as described previously.^39^^,^^42^ A head holder and recording chamber (Gray Matter Research) were fixed to the skull under general anesthesia and aseptic conditions. We located the anatomical position of the amygdala from bone marks on coronal and sagittal radiographs in reference to the stereotaxically implanted chamber.^69^ We recorded the activity of single amygdala neurons from extracellular positions while the animals performed the task, using standard electrophysiological techniques including on-line visualization and threshold discrimination of neuronal impulses on oscilloscopes. We aimed to record representative neuronal samples from the lateral, basolateral, basomedial and centromedial amygdala. A stainless-steel tube (0.56 mm outer diameter) guided a single tungsten microelectrode of 0.125 mm diameter and 1- to 5-MΩ impedance (FHC Inc.) through the dura and assured consistent targeting of subcortical structures. We advanced the microelectrode vertically in the stereotaxic plane using a hydraulic micromanipulator (MO-90; Narishige, Tokyo, Japan). Neuronal signals were amplified, bandpass filtered (300 Hz–3 kHz), and monitored online with oscilloscopes. Somatodendritic discharges from single amygdala neurons were distinguished from background noise and other neurons using a time threshold window discriminator (WD-95; Bak Instruments), which produced a 1.0-ms-long standard transistor-transistor logic pulse for each neuronal impulse that helped in the online inspection of neuronal recordings. Behavioral data, digital signals from the impulse window discriminator, and analogue eye position data were sampled at 2 kHz on a laboratory computer with custom MATLAB (Mathworks Inc.) code. We recorded analogue impulse waveforms at 22 kHz with a custom recording system and sorted them offline for data analysis, using cluster-cutting and principal component analysis (Offline sorter; Plexon), which provided the database for the present manuscript. We used one electrode per session and recorded between 1 and 3 neurons per session (monkey A: 1.41 ± 0.05; monkey B: 1.32 ± 0.08).

During recordings, we sampled activity from about 1,000 amygdala neurons and recorded and saved the activity of neurons that appeared to respond to any task event during online inspection of several trials. Thus, we aimed to identify task-responsive neurons but did not preselect neurons based on specific response characteristics. This procedure resulted in a database of 510 neurons (233 neurons in the main task, 205 neurons in the four-objects task, 72 neurons in the two-juices task), which we analyzed statistically. Statements about the number of neurons showing specific effects are made with reference to these task-related neurons. The number of neurons is similar to those reported in previous studies on primate amygdala.

#### Reconstruction of neuronal recording sites

Following completion of all data collection, the animals received an overdose of pentobarbital sodium (90 mg/kg iv) and were perfused with 4% paraformaldehyde in 0.1 M phosphate buffer through the left ventricle of the heart. We reconstructed the neuronal recording positions from 50-μm-thick, stereotaxically oriented coronal brain sections stained with cresyl violet based on electrolytic lesions (15–20 μA, 20–60 s, made in one animal) and lesions by cannulas that were placed to demarcate recording areas, by recording coordinates for individual neurons noted during experiments, and in reference to other brain structures with known electrophysiological signatures recorded during experiments (internal and external globus pallidus, substantia innominata).^70^ We assigned recorded neurons to amygdala subnuclei with reference to a stereotaxic atlas^71^ at different anterior-posterior positions (the figures show locations of recorded neurons collapsed over anterior-posterior levels). In the main task, we recorded 93 neurons from the lateral amygdala (52 dorsal, 41 ventral), 94 neurons from the basolateral amygdala, 17 neurons from the basomedial (also termed accessory basal) amygdala and 29 neurons from the centromedial amygdala (Figure S3E). Neurons were not recorded simultaneously from different nuclei. We assigned recorded neurons to dorsal and ventral portions of the lateral nucleus in reference to a previous paper.^49^ A neuron was classified as belonging to dLA or vLA if its recording position was consistent with the lateral nucleus, as determined by reconstruction from histology and stereotaxic coordinates,^71^ and located either in its dorsal or ventral half, respectively in reference to a previous study.^49^ We made no attempt to distinguish dorsal intermediate and ventral intermediate divisions of the lateral nucleus.

#### Main choice task

Two monkeys performed in a reward-based choice task with sequentially presented choice options under computer control (Figure 1C). Our goal was not to establish whether the animals used a general view-based behavioral strategy, but rather to study the activity patterns of amygdala neurons during this task. The animal sat in a primate chair (Crist Instruments) with a horizontally mounted touch screen for stimulus display placed in front of them (EloTouch 1522L 15’; Tyco). On each trial, the animal made a choice between two sequentially presented options. Each option consisted of a visual ‘object’ (fractals, abstract images, photographs of natural objects such as flowers) presented in central position on the computer monitor overlaid by a small bar stimulus. We used two visual objects in the main task. Different objects were associated with specific reward probabilities that varied across the testing session without notification. Different bar heights cued different reward magnitudes chosen randomly on each trial. To maximize reward, the animals were required to learn and track the (uncued) reward probabilities associated with the different objects and combine these probability estimates with the trial-specific cued reward magnitudes for the different objects. Reward probabilities varied in blocks of 15–40 trials and were pseudorandomly chosen for each object from the following set: 0, 0.15, 0.35, 0.5, 0.65, 0.75, 0.85, 1.0. Reward magnitudes varied randomly on each trial and were chosen from the following set: 0.25 mL, 0.4 mL, 0.65 mL. The specific reward probabilities and magnitudes were chosen based on pre-testing to ensure that the animals maintained high motivation during the task while at the same time providing sufficient variation in choices and modeled object values. Importantly, reward magnitudes were only cued transiently, during sequential object presentation, but not in the period when the animals indicated their choice with a saccade. This transient presentation of reward-magnitude cues was designed to encourage the animals to make a decision during sequential object viewing, rather than in the later saccade period. A computer-controlled solenoid valve delivered juice reward from a spout in front of the animal’s mouth. On each completed trial, the acting animal received one of two outcomes: on ‘rewarded’ trials, a liquid reward corresponding to the cued reward amount in ml was delivered whereas on ‘non-rewarded’ trials, a small reward of 0.05 mL was delivered. We found that a small reward instead of non-reward on ‘unrewarded’ trials ensured that the animals maintained high motivation. During the periods of neurophysiological recordings and behavioral testing, the animals received their main liquid intake during task performance, supplemented by additional liquid after the testing sessions if required. The animals had free access to foods in their home cage.

Each trial started when the background color on the touch screen changed from black to gray. To initiate the trial, the monkey was required to place his hand on an immobile, touch-sensitive key. Presentation of the gray background was followed by presentation of an ocular fixation spot (1.3° visual angle). On each trial, the animal was then required to fixate this spot within 4° for 500 ms. Following 500 ms of central fixation, a first choice cue (‘object’) and overlaid bar stimulus appeared centrally for 500 ms and were followed, after cue-offset, by a 500 ms inter-stimulus interval, which was then followed by a second choice cue and overlaid bar stimulus shown for 500 ms followed by another 500 ms inter-stimulus interval. The reward magnitude cue covered 18.75 percent of the underlying image. The two objects could have the same reward magnitude on a given trial, as determined by random permutation. Animals A and B performed 108 and 36 sessions, respectively. We used new objects in each session in each animal resulting in 108 image sets for animal A and 36 image sets for animal B. Following sequential presentation of these individual choice objects and overlaid bar stimuli, the two objects reappeared simultaneously on the left and right side of the monitor (determined pseudorandomly); importantly, the magnitude-bar stimuli did not reappear. Thus, the separate presentation of the first and second reward-magnitude cue, and their transient presentation during sequential viewing precluded simultaneous magnitude comparison. After 100 ms, the fixation spot disappeared, indicating that the monkey was no longer required to fixate the spot and was allowed to make his choice by fixating the object on the left or right for 500 ms. The monkey was allowed to freely look back and forth between the objects for 2,000 ms and in that period could make a choice at any time by fixating the chosen object for 500 ms. Once the monkey’s choice was registered, the unchosen object disappeared and after a delay of 500 ms, the chosen object also disappeared and a liquid reward was given depending on the scheduled reward probability and magnitude for the chosen option. Reward delivery was followed by a trial-end period of 1,000–2,000 ms which ended with extinction of the gray background. The next trial started after an inter-trial interval of 2,000–4,000 ms (drawn from a uniform random distribution). A recording session for a given neuron would typically last 150 trials.

Possible errors in performance included failure to make contact with the touch-sensitive key before the trial, key release before saccade choice, failure to fixate a choice object for 500 ms during the choice period, failure to fixate the central fixation spot at trial start or fixation break in the period between initial fixation and disappearance of fixation spot. Errors led to a brief time out (3,000 ms) with a black background and then trial repetition. Task performance was typically interrupted after three consecutive errors. The animals were required to fixate the fixation spot and the objects until the choice targets were presented in left-right arrangement. Fixation was continually monitored by the task program during all of these periods and fixation breaks resulted in an error trial. The animals were required to place their hand on a touch-sensitive key to initiate each trial and keep their hand in place on the key until trial completion.

Task training of the animals progressed as follows. Following habituation to the laboratory environment and experimental set-up, we trained the monkeys in successive steps to drink liquid reward from the spout, place their hands on a touch key and hold the touch key for increasingly longer periods to receive reward, to view different visual conditioned stimuli that resulted in reward delivery, to touch and choose between visual stimuli on a touch screen, to choose between visual stimuli based on fixed stimulus-associated reward probability or cued reward magnitude, to choose between visual stimuli under conditions of varying reward probability or magnitude, to choose between stimuli that varied in both reward probability and reward magnitude, to perform the task under head-fixation, to perform the task under gradually increasing visual fixation requirements including saccade choices. We progressed from task training to recording once the animals were implanted with recording chambers and when their performance had reached an asymptotic level. These training periods, including development of the tasks, lasted approximately 24 and 18 months for animals A and B.

Stimuli and behavior were controlled using custom MATLAB code (The Mathworks) and Psychophysics toolbox (version 3.0.8). The laboratory was interfaced with data acquisition boards (NI 6225; National Instruments) installed on a PC running Microsoft Windows 7.

#### Control task with four objects

We recorded amygdala neurons in a separate task with four different visual objects, organized in two object sets of two objects each that changed across trial blocks. These data helped determine whether view-based choice signals would generalize over a larger number of visual objects. Recordings were performed from the same two monkeys as in the main task. The task structure was simpler compared to the main task as reward value derived only from changing object-reward probabilities without additional reward magnitude information (no superimposed magnitude cues were used in this task). Moreover, the data were recorded in a social context in which two monkeys sat opposite to each other and took turns making choices for separate visual object sets.^42^ Object sets switched half-way through a given testing session, allowing us to analyze the recorded monkey’s neuronal data in relation to two object sets. One object within a pair was associated with a reward probability of 0.85, whereas the other object was associated with a reward probability of 0.15. Reward probabilities reversed between objects after blocks of typically 25–35 trials per animal. On each completed trial, the acting animal received one of two outcomes: on ‘rewarded’ trials, a liquid reward of 0.8 mL was delivered whereas on ‘non-rewarded’ trials, a small reward of 0.05 mL was delivered. The observer animal did not receive any reward. A typical recording of one neuron would consist of about 200 choice trials.

Each trial started when the touch-screen background color changed from black to gray. To initiate a trial, both monkeys were required to place their hand on an immobile, touch-sensitive key (each animal had its own touch key). Following presentation of the gray background, we presented an ocular fixation spot (1.3° visual angle). On each trial, the recorded animal was required to fixate the spot within 4° for 500 ms. Following 500 ms of central fixation, a first choice cue appeared centrally for 350 ms and was followed, after cue-offset, by a 350 ms inter-stimulus interval, which was then followed by a second choice cue shown for 350 ms and another 350 ms inter-stimulus interval. As in the main task, the two objects then reappeared simultaneously on the left and right side of the monitor (determined pseudorandomly). After 100 ms the fixation spot disappeared, two blue rectangles appeared below the choice objects and the acting animal was required to touch one of the object-associated blue rectangles within 1.5 s to make its choice. The unchosen object then disappeared and after a delay of 500 ms, the chosen object also disappeared and a liquid reward was given to the acting animal. Reward delivery was followed by a trial-end period of 1,000–2,000 ms which ended with extinction of the gray background. The next trial started after an inter-trial interval of 2,000–4,000 ms (drawn from a uniform random distribution). The roles of acting and non-observing animal reversed after every correct trial. Behavioral data and neuronal from this task were previously reported in an investigation of the neuronal processing of the social aspects of the task^42^; here we re-analyzed the neuronal data on the recorded monkeys’ trials to test for the presence of view-based choice neurons.

#### Control task with different reward types

We recorded amygdala neurons in a control task with two physically different liquid rewards to distinguish neuronal coding of view-based choice from choice signals related to different reward types. Recordings were performed in one of the animals tested in the main task. The design and trial structure of the ‘two-juices task’ was similar as for the main task except that value derived from the cued reward magnitude (as in the main task) and variations in reward type, but not from changing reward probabilities. Throughout a testing session, the two distinct visual objects were associated with delivery of two distinct physical liquid rewards. The liquid rewards included water and diluted fruit juices (blackcurrant, apple, orange, lemon, alphonso mango, pomegranate, peach). To dissociate effects related to visual objects and rewards, associations between visual objects and rewards typically changed twice in each recording session. In the first 60 trials of a session, the monkey would choose between objects A and B that predicted different rewards, e.g., apple juice and lemon juice, respectively. The object-reward association would then reverse without notification and for the next 60 trials, the monkey would choose between objects A and B that now predicted lemon and apple juice, respectively. In separate training sessions without reward magnitude variation, we found that the monkey adapted typically in less than three trials to changes in object-reward association by switching his choices from one visual object to the alternative object to track the preferred reward. The trial structure was the same as in the main task, except that presentation of each option lasted for 350 ms (rather than 500 ms as in the main task), followed by a 350 ms inter-stimulus interval. The monkey indicated his choice by a saccade to the preferred object. As in the main task, reward magnitudes were only transiently cued during sequential option presentation but not during the saccade choice period.

### Quantification and statistical analysis

#### Behavioral data analysis

##### Reinforcement learning model

To describe the animals’ behavior in the main task, and to derive trial-by-trial measures of object values for neuronal analysis, we fitted reinforcement-learning (RL) models to the animals’ choices. The best-fitting model (‘Reversal RL’, see Table S1) accounted for the reversal-learning nature of the task by updating both the value of the chosen and unchosen option on each trial, as done in previous studies with reward-reversal learning tasks.^42^^,^^72^ Object values in this model were updated as follows (Equation 1):

(Equation 1) $$V_{A}^{t+1}=V_{A}^{t}+\alpha \left(R^{t}-V_{A}^{t}\right)$$

$$V_{B}^{t+1}=V_{B}^{t}+\alpha \left({-R}^{t}-V_{B}^{t}\right)$$

with $V_{A}^{t}$ as the expected value of object A on trial t, $R^{t}$ as reward (coded as 0 or 1 for small and large reward, respectively), $R^{t}-V_{A}^{t}$ as prediction error between reward $R^{t}$ and expected value $V_{A}^{t}$ on trial t, α as free-parameter learning rate and $V_{A}^{t+1}$ as the updated expected object value for the next trial, and corresponding variables for the alternative object B. Note that the prediction error for object B, ${-R}^{t}-V_{B}^{t}$ , involved updating the value for object B in the opposite direction as for object A. This model is a variant of standard reinforcement learning as it updates additionally the value of the unchosen option. The object choice on each trial was determined by the softmax rule^73^ (Equation 2):

(Equation 2) $$P\left(A\right)=\frac{1}{\left(1+\exp\left(-\beta \left(V_{A}^{t}-V_{B}^{t}\right)\right)\right)}$$

with P(A) as choice probability for object A and β as the free-parameter inverse temperature, which reflects the degree of stochasticity in the animal’s choices.

We estimated the model’s free parameters by fitting the model to the trial-by-trial record of choices and rewards within each session, separately for each session and separately for the two animals. Model fitting was performed using a maximum likelihood procedure with the Nelder–Mead search algorithm (implemented by the MATLAB function ‘fminsearch’).

We compared several alternative reinforcement-learning models with the results of the model comparison shown in Table S1. The additional models tested include: (1) a basic reinforcement-learning model formulated as above but without updating the value of the unchosen option (‘Basic RL’ in Table S1), (2) a model formulated as the basic model but using two separate learning rates for rewarded and unrewarded trials (‘Basic Model, two learning rates’), a model formulated as our main model (Equation 2) but with separate learning rates for rewarded and unrewarded trials (‘Reversal RL, two learning rates’), a Pearce-Hall model in which the learning rate depended on the unsigned reward prediction error (‘Pearce-Hall), a model formulated as our main model (Equation 2) but with a learning rate that depended on the unsigned reward prediction error (‘Pearce-Hall, reversal learning’), a Pearce-Hall model using separate learning rates for rewarded and unrewarded trials (‘Pearce-Hall, reversal learning, two learning rates’). The best-fitting model was identified using Akaike Information Criterion and Bayesian Information Criterion (Table S1).

For the optimality analysis in Figures S1G and S1H, we simulated the reversal-learning model (Equation 1) by systematically varying the learning rate and inverse temperature free parameters, and included free parameters that determined the weight assigned to model-derived probability estimates and cued reward magnitudes. Simulations were performed using the block-wise object-reward probabilities used in each experimental session. The simulation was repeated 100 times for each experimental session and each combination of the free parameters. The learning rate was varied between values of 0 and 1 with a step size of 0.01; the inverse temperature was varied between values of 0 and 5 with a step size of 0.05. This procedure resulted in a distribution of reward magnitudes that the reinforcement learner obtained across simulated trials for each combination of free parameter values (Figure S1G, shown for simulations with equal probability and magnitude weighting). For Figures S1H, we performed the above simulations without modeling the effect of reward magnitudes.

##### Mixed-effects multinomial logistic regression

We used mixed-effects multinomial logistic regression analysis (*fitglme* function, MATLAB) to model the animals' trial-by-trial choices across testing sessions. Specifically, we modeled choices for the first- or second-presented option separately for each animal and specified the categorical session number (*Session*) as the group variable to account for session-by-session variations (random effects). We adopted the global model in which we estimated both the main effects and random effects of all the relevant regressors. The response variable was the dichotomous first (FirstChosen=1) or second (FirstChosen=0) trial-by-trial choice, collected from $S_{k}$ sessions in monkey k ($S_{k}\in N,k=1,2$). In the framework of generalized linear mixed models with logit function as the link function, the logistic regression model can be specified as follows:

$$logit\left(\pi_{ij}^{L}\right)=\log\left(\frac{\pi \left({FirstChosen}_{ij}=1\right)}{\pi \left({SecondChosen}_{ij}=0\right)}\right)=x_{ij}^{\prime }\beta +z_{ij}^{\prime }u_{i}+\varepsilon_{ij},\varepsilon_{ij}∼Normal\left(0,\sigma^{2}\right)$$

where $\pi_{ij}^{L}$ denotes the probability of choosing the first option in the j th trial of session i ($j=1,2,\ldots ,T_{i}\in N;T_{i}=$ the total number of trials in session i); $x_{ij}$ is a vector of trial-by-trial predictors (fixed-effect regressors; see below) and $z_{ij}$ is vector of trial-by-trial predictors nested in $x_{ij}$, and the effects of these predictors vary across sessions (random-effect regressors). The model estimated the coefficients of fixed-effect regressors, β, and the session-wise variations of the random-effect regressors, $u_{i}$. The estimated first-second choice responses, $p_{ij}^{L}$, were derived by reverse logit function conditional on the session-wise random effects ($u_{i}$), and the session-wise regression coefficients ($\eta_{i}$) were derived from the fixed-effect coefficients (β) and the session-wise calibration terms ($u_{i}$).

$$p_{ij}^{L}=P\left(FirstChosen=1|u_{i}\right)=\frac{\exp\left(x_{ij}^{\prime }\beta +z_{ij}^{\prime }u_{i}\right)}{1+\exp\left(x_{ij}^{\prime }\beta +z_{ij}^{\prime }u_{i}\right)}\in \left[0,1\right],\eta_{i}=\beta +u_{i}$$

In the main model (Table S2), we included the following regressors. Importantly, we specified the categorical session number (*Session*) as the group variable to address session-wise variations of nutrient sensitivities as follows (Equation 3),

(Equation 3) $$logit\left(FirstChosen\right)=\beta_{0}+\beta_{1}\times FirstLeft$$

$$+\beta_{2}\times FirstRM+\beta_{3}\times SecondRM+\beta_{4}\times FirstProb+\beta_{5}\times SecondProb\;|\;Session$$

where FirstLeft indicated whether the first option was subsequently shown on the left during the saccade-choice period (1, if the first option was shown left; 0 if the first option was shown right), FirstRM indicated the trial-specific reward magnitude associated with the first option; SecondRM indicated the trial-specific reward magnitude associated with the second option, and FirstProb−SecondProb indicated the difference in reward probability between the first and second option.

The mixed-effect model defined above served to quantify the statistical significance of the different variables in a comprehensive manner using the full dataset across all sessions.

To define object values in individual sessions as regressors for neuronal analysis, we used the following model (Equation 4) that we fit to data in individual sessions,

(Equation 4) $$logit\left(ObjectA\;Chosen\right)=\beta_{0}+\beta_{1}\times ObjectA\;First$$

$$+\beta_{2}\times \left(ObjectA\;RM-ObjectB\;RM\right)+\beta_{3}\times \left(ObjectA\;Prob-ObjectB\;Prob\right)$$

where ObjectAChosen indicated whether object A (rather than object B) was chosen on a given trial, ObjectAFirst indicated whether object A was shown left on a given trial, ObjectARM and ObjectBRM indicated the reward magnitudes for object A and B on a given trial, and ObjectAProb and ObjectBProb indicate the reward probabilities on a given trial. Results from this model are shown in Table S2, based on means (±SEM) and t test statistics across sessions. We used the regression coefficients $\beta_{2}$ and $\beta_{3}$ from this model to define object value as follows (Equation 5).

(Equation 5) $$Object\;value=\beta_{2}\times RM+\beta_{3}\times Prob$$

This definition accounted for any animal-specific and session-specific weighting of reward magnitude and probability that occurred in a given testing session. Object values derived from this equation were used as session-specific value-regressors in all neuronal analyses, except where otherwise noted (e.g., in cases in which we used only the reward-magnitude value component as regressor for specific purposes).

##### Eye data processing

We monitored the animals’ eye positions using an infrared eye tracking system at 125 Hz (ETL200; ISCAN) placed next to the touchscreen. Before each recording session, we calibrated the eye tracker during a fixation task with a moving fixation spot that the animal had to follow. During recordings, accuracy of calibration of the eye tracker was regularly checked and if necessary recalibrated. The monkey’s head was slightly tilted forward (∼10°) for a better view of the touchscreen. We assessed eye position in a plane in front of the monkey’s eyes, followed by a transformation to the horizontal touchscreen plane.^42^ We then determined whether and when a fixation occurred. We defined a fixation when eye velocity was below 25% of its statistical standard deviation for more than 60 ms. For analysis of fixations in specific task-related time windows, we excluded fixations that occurred within the first 100 ms of stimulus onset to remove anticipatory fixations. We selected fixations that met the above criteria.

##### Neuronal data analysis

We counted neuronal impulses for each neuron on correct trials in fixed time windows relative to different task events focusing on the following non-overlapping task epochs: 500 ms after fixation spot before cues (Fixation), 500 ms after onset of first cue (i.e., first choice object), 500 ms after offset of first cue, 500 ms after onset of second cue, 500 ms after offset of second cue, 500 ms after onset of choice targets. We did not observe systematic differences in activity patterns between animals in preliminary analyses; therefore, we pooled data from both animals for subsequent analyses.

Our analysis strategy was as follows. We used fixed-window and sliding-window linear and multi-linear regression analyses to identify neuronal responses related to specific variables. For fixed-window analyses, we first identified task-related object-evoked responses by comparing activity during object presentation (first and second cue period) to a baseline control period (before appearance of fixation spot) using the Wilcoxon test (p < 0.005, Bonferroni-corrected for multiple comparisons). A neuronal response was classified as task-related if it was significantly different from activity in the control period (the pre-fixation period on each trial of the main task). We used a multiple linear regression model to test whether neuronal activities were significantly related to specific task variables (p < 0.05, t test on regression coefficient) while including other relevant variables as covariates. We also used sliding-window multiple regression analyses with a 200-ms window that we moved in steps of 20 ms across each trial (without pre-selecting task-related responses). Sliding-window analyses tested for dynamic coding of different task-related variables over time within trials and also confirmed that our results did not depend on the pre-selection of task-related responses or definition of fixed analysis windows. To determine statistical significance of sliding-regression coefficients, we used a permutation-based approach as follows. For each neuron, we performed the sliding-window regression 1,000 times using trial-shuffled data and determined a false positive rate by counting the number of consecutive sliding-windows in which a regression was significant with p < 0.05. We found that less than 5% of neurons with trial-shuffled data showed more than nine consecutive significant analysis windows. Accordingly, we classified a sliding-window analysis as significant if a neuron showed a significant (p < 0.05) effect for more than nine consecutive 20-ms windows. Statistical significance of regression coefficients was determined using t test; all tests performed were two-sided. Additional population decoding, described below, examined independence of our findings from pre-selection of task-related responses and served to assess information about specific task variables contained in the neuronal population.

We performed our regression analysis in the framework of the general linear model (GLM) implemented with the MATLAB function (*glmfit*). Neuronal responses were tested with the following regression models:

GLM 1 (Equation 6): This GLM served to identify value-coding neurons and distinguish object-value from view-based value signals. It also served to derive regression coefficients for Figures 2G and 5B. To distinguish different types of value signals, we adapted a method of classification of neuronal value responses based on the angle of regression coefficients.^74^^,^^75^ This classification method is ‘axis-invariant’ as it is independent of the axis choice for the regression model, i.e., whether the model includes separate variables for both object values or view-based values.^74^ For the main analysis reported in Figure 2G, we calculated neuronal activity in a 500-ms fixed window after onset of the first choice object. This analysis constituted a strict test of neuronal value coding in the absence of value comparison (which could only commence once the second object had been viewed).

(Equation 6) $$y=\beta_{0}+\beta_{1}\;\left(Object\;value\right)+\varepsilon$$

with y as the neuronal activity in response to the presentation of a specific choice object (at first-viewed object), Objectvalue as the value of the viewed object (A or B), calculated using Equation 5. Standardized regression coefficients (betas) obtained from this model were used for Figure 2G and defined as x_i_(s_i_/s_y_), *x*_*i*_ being the raw slope coefficient for regressor *i*, and *s*_*i*_ and *s*_*y*_ the standard deviations of independent variable *i* and the dependent variable, respectively. We report also the results from regressions that combined neuronal responses for both objects at the first cue, examined neuronal responses at the second cue, collapsed across first and second cue periods, and a model that included a chosen-value covariate. The identification of object-value and view-based value neurons was robust across these different analysis approaches.

Using this method, a neuronal response was categorized as value-related if it showed a significant overall model fit (p < 0.05, F test). For responses with significant model fit, we plotted the magnitude of the beta coefficients (standardized slopes) of the two object-value regressors on an x-y plane. We followed a previous study^74^ and divided the coefficient space into eight equally spaced segments of 45° to categorize neuronal responses based on the polar angle in this space of regression coefficients (Figure 2G). We categorized responses as coding object value if their coefficients fell in the segments pointing toward 0° or 180° (object value A) or toward 90° or 270° (object value B), indicating a relationship to only one of the two values. We categorized responses as coding view-based value if their coefficients fell in the segments pointing toward 135° or 315° or in the segments pointing toward 45° or 225°, indicating a relationship to both object values. The joint presence of both object-value and view-based value neurons was also confirmed with a separate stepwise regression approach (Figure S2).

For the analysis of neuronal activity during the saccade-choice period (Figure 2J), we fitted the above model (Equation 6) to neuronal activity in 300-ms windows aligned to the onset of a fixation of one of the two choice objects. We analyzed fixations in the period from the onset of object-choice targets until 500 ms after target offset. We selected fixations that fell into a region of interest for the left or right object. For Figure 2J, we calculated neuronal activity in a 600-ms window starting 100 ms before onset of fixation to 500 ms after fixation onset in pre-selected neurons that encoded view-based value (N = 61) or object-A value (N = 32) from Equation 6 and plotted the time course of z-normalized neuronal activity aligned to the onset of first, and second fixations, split by value (median split).

GLM 2 (Equation 7): This GLM served to identify neurons encoding view-based choice, while controlling for other variables. It also served to derive partial-R^2^ values (coefficients of partial determination) from for Figures 3M, 3N, 5D, S9A, S9B, and S10H.

(Equation 7) $$y=\beta_{0}+\beta_{1}\;\left(ViewChoice\right)+\beta_{2}\;\left(ObjectChoice\right)+\beta_{3}\;\left(ObjectView\right)$$

$$+\beta_{4}\;\left(FirstValue\right)+\beta_{5}\;\left(SecondValue\right)+\beta_{6}\;\left(ChosenValue\right)$$

$$+\beta_{7}\;\left(ObjectALeft\right)+\beta_{8}\;\left(LeftChosen\right)+\varepsilon$$

with y as the neuronal activity in a 200-ms sliding window, aligned to the onset of the first choice cue and moved in 20-ms steps from 500 ms before the onset of the first cue until 500 ms after the onset of the choice targets, ViewChoice as view-based choice, defined as choice for the first-viewed or second-viewed object on a given trial (coded as 1 and 0, respectively), ObjectChoice as the choice for object A or object B on a given trial (coded as 1 and 0, respectively), ObjectView as the viewing order for objects A and B on a given trial (coded as 1 for A-then-B and 0 for B-then-A), FirstValue as the value for the first-viewed object on a given trial (derived from Equation 6), SecondValue as the value for the second-viewed object on a given trial (derived from Equation 6), ChosenValue as the value for the chosen object on a given trial, ObjectALeft as the left-right cue position for object A (coded as 0 for right and 1 left), LeftChosen as the left-right choice (coded as 0 for right chosen and 1 for left chosen).

GLM 3 (Equation 8): This GLM served to identify neurons encoding view-based choice, while controlling for reward probability and reward magnitude (instead of the integrated values).

(Equation 8) $$\begin{array}{ll} y= & \beta_{0}+\beta_{1}\;\left(ViewChoice\right)+\beta_{2}\;\left(ObjectChoice\right)+\beta_{3}\;\left(ObjectView\right)\\ & +\beta_{4}\;\left(FirstProb\right)+\beta_{5}\;\left(SecondProb\right)+\beta_{6}\;\left(FirstMag\right)+\beta_{7}\;\left(SecondMag\right)\\ & +\beta_{8}\;\left(ChosenProb\right)+\beta_{9}\;\left(ChosenMag\right)+\beta_{10}\;\left(ObjectALeft\right)\\ & +\beta_{11}\;\left(LeftChosen\right)+\varepsilon \end{array}$$

with FirstProb and SecondProb as the probability as the reward probability of the first-viewed and second-viewed object on a given trial (derived from Equation 1), respectively; FirstMag and SecondMag as the cued reward magnitude of the first-viewed and second-viewed object on a given trial, respectively; and ChosenProb and ChosenMag as the probability and magnitude for the chosen object on a given trial, respectively.

GLM 4 (Equation 9): This GLM served to derive value-regression coefficients for the analyses shown in Figures 3O, 5B, S9C, S9F, S9I, S9J, S9M, and S9O.

(Equation 9) $$y=\beta_{0}+\beta_{1}\;\left(Value\right)$$

with y as neuronal activity in a 500-ms fixed window after onset of the first or second choice cue and Value as the value of the first or second viewed choice option. Importantly, as the analysis examines relationships between value regression coefficients, we used only the reward-magnitude component of the value of each choice object, to remove any intrinsic anti-correlation between values derived from the reinforcement-learning model. Because the reward magnitudes between the first and second choice option were uncorrelated in each session (see Figure S9), the analysis was not biased toward detecting a positive or negative relationship between value coefficients.

GLM 5 (Equation 10): This GLM served to identify neurons encoding view-based choice in the four-objects choice task, while controlling for other variables. It also served to derive partial-R^2^ values for Figures S9G and S9H.

(Equation 10) $$\begin{array}{ll} y= & \beta_{0}+\beta_{1}\;\left(ViewChoice\right)\\ & +\beta_{2}\;\left(ObjectAChoice-ObjectBChoice\right)+\beta_{3}\;\left(ObjectCChoice-ObjectDChoice\right)\\ & +\beta_{4}\;\left(ObjectAView-ObjectBView\right)+\beta_{5}\;\left(ObjectCView-ObjectDView\right)\\ & +\beta_{6}\;\left(FirstValue\right)+\beta_{7}\;\left(SecondValue\right)+\beta_{8}\;\left(ChosenValue\right)+\varepsilon \end{array}$$

with ObjectAChoice−ObjectBChoice indicating choice for object A or object B in the trial block in which these objects were shown (coded as 1 for object A chosen, −1 for object B chosen, and 0 otherwise), ObjectCChoice−ObjectDChoice indicating choice for object C or object D in the trial block in which these objects were shown (coded as 1 for object C chosen, −1 for object D chosen, and 0 otherwise), ObjectAView−ObjectBView indicating whether object A or object B was shown first or second in the trial block in which these objects were shown (coded as 1 for object A first, −1 for object B first, and 0 otherwise), ObjectCView−ObjectDView indicating whether object C or object D was shown first in the trial block in which these objects were shown (coded as 1 for object C first, −1 for object D first, and 0 otherwise), and all other regressors as specified in GLM 2. We omitted regressors for cue position and left-right choice to reduce the number of variables in the model and because these regressors were not relevant to the main task periods analyzed with this model; including these regressors did not alter the number of identified view-based choice neurons.

GLM 6 (Equation 11): This GLM served to identify neurons encoding view-based choice in the two-juices choice task, while controlling for other variables. It also served to derive partial-R^2^ values for Figures S9D and S9E.

(Equation 11) $$\begin{array}{ll} y= & \beta_{0}+\beta_{1}\;\left(ViewChoice\right)+\beta_{2}\;\left(ObjectAChoice\right)+\beta_{3}\;\left(JuiceAChoice\right)\\ & +\beta_{4}\;\left(ObjectAView\right)+\beta_{5}\;\left(JuiceAfirst\right)+\beta_{6}\;\left(FirstValue\right)\\ & +\beta_{7}\;\left(SecondValue\right)+\beta_{8}\;\left(ChosenValue\right)+\beta_{9}\;\left(ObjectALeft\right)\\ & +\beta_{10}\;\left(LeftChosen\right)+\varepsilon \end{array}$$

with JuiceAChoice as choice for juice A (coded as 1 for juice A choice and 0 for juice B choice), JuiceAFirst indicating trials on which juice A was shown first (coded as 1 for juice A first and 0 for juice B first), FirstValue and SecondValue as the value of the first and second option defined by the cued reward magnitude, respectively, and all other definitions as in GLM2.

##### Normalization of population activity

To normalize activity from different amygdala neurons, we subtracted from the impulse rate in a given task period the mean impulse rate of the pre-fixation control period and divided by the standard deviation of the control period (*Z* score normalization). We also distinguished neurons that showed positive relationships or negative relationships with a given variable, based on the sign of the regression coefficient, and sign-corrected responses with a negative relationship. Normalized data were used for Figures 2J, 3E, S10E, and S12B, and all decoding and RSA analyses.

##### Normalization of regression coefficients

Standardized regression coefficients were defined as x_i_(s_i_/s_y_), *x*_*i*_ being the raw slope coefficient for regressor *i*, and *s*_*i*_ and *s*_*y*_ the standard deviations of independent variable *i* and the dependent variable, respectively. Standardized regression coefficients were used for Figures 2G and 3O, 5B, S9C, S9F, S9I, S9J, S9M, and S9O.

##### Population decoding

We used a SVM classifier to quantify information about task-related variables contained in neuronal population activity in defined task periods, following previous neurophysiological studies.^42^^,^^75^^,^^76^ The SVM classifier was trained to find a linear hyperplane that best separated patterns of neuronal population activity defined by a given grouping variable (e.g., high vs. low value, choice for currently viewed vs. last-viewed object, choice for object A vs. object B); the different levels of a given grouping variable are referred to as ‘groups’ in the following text. We also used a NN classifier, which assigned each trial to the group of its nearest single-trial neighbor in a space defined by the distribution of impulse rates for different levels of the grouping variable using the Euclidean distance. The NN classifier in particular can be described as biologically plausible, in the sense that a downstream neuron could perform a similar classification by comparing the input on a given trial, provided by a neuronal population-activity vector, with a stored synaptic-weight vector. Both classifiers performed qualitatively similar but SVM decoding was typically more accurate.

To prepare data for decoding, we aggregated z-normalized trial-by-trial impulse rates of the separately recorded amygdala neurons from specific task periods into pseudo-populations. We used all recorded neurons that met inclusion criteria for a minimum trial number, without pre-selecting for coding a specific variable. Depending on the variable used for decoding, we only included neurons in the decoding analyses that had a minimum number of 5, 10 or 15 trials per group for which decoding was performed; we confirmed that results were robust to changes in this minimum trial number. We created two *n* by *m* matrices with *n* columns determined by the number of neurons and *m* rows determined by the number of trials. We defined two matrices, one for each group for which decoding was performed, using the following different groupings. For object-value decoding, we defined separate groups for low and high object value, determined for each neuron by calculating value terciles. (We obtained very similar results by repeating the decoding analyses based on median-split.) For choice decoding, we defined two separate groups depending on either the view-based choice (currently viewed or last-viewed chosen) or object choice (A or B) on each trial. Accordingly, each cell in a matrix contained the normalized impulse rate from a single neuron on a single trial measured for a given group. Because neurons were not simultaneously recorded, we randomly matched up trials from different neurons for the same group in the matrix used for decoding, and repeated the decoding analysis with different random trial matching (‘within-group trial matching’) 150 times for SVM and 500 times for NN. We found these numbers of repetitions produced stable classification results and confirmed robustness with respect to changes in this number. (Our approach likely provides a lower bound for decoding performance because it does not account for potential contributions from cross-correlations between neurons; investigation of cross-correlations would require data from simultaneously recorded neurons.)

We quantified decoding accuracy as the percentage of correctly classified trials, averaged over all decoding analyses for different random within-group trial matchings. We used a leave-one-out cross-validation procedure: a classifier was trained to learn the mapping from impulse rates to groups on all trials except one test trial; this remaining trial was then used for testing the classifier and the procedure repeated until all trials had been tested. We obtained similar results when splitting data into 80% training trials and 20% test trials. We used a rank-sum test to compare the classification performance against performance obtained from data in which the group labels were randomly shuffled 1,000 times. We implemented SVM decoding in MATLAB (Mathworks, Natick, MA) using the *svmtrain* and *svmclassify* functions with a linear kernel and the default sequential minimal optimization method for finding the separating hyperplane. The NN decoding was implemented in MATLAB with custom code. Statistical significance was determined by comparing vectors of percentage correct decoding accuracy between real data and randomly shuffled data (in which group labels had been shuffled) using the rank-sum test.

For cross-decoding analyses shown in Figures 2H, 3F, 5C, S7, S9P, and S14, we trained the classifier on data recorded in one particular experimental condition (e.g., object-viewing sequence A-then-B) and tested the classification performance on data recorded in a different condition (e.g., B-then-A). We previously used a similar approach in a social task, to test whether amygdala neurons encoded task-related variables in a common reference frame across self and other.^42^ Here, we used the same approach to investigate common reference frames for value and choice signals across objects and rewards. For the rightmost plot in Figure 2H we trained the classifier to decode value during the first-cue period and then tested the classifier performance to decode choice (for the currently viewed vs. last viewed cue) during the second-cue period. For Figure 2H, we performed decoding on N = 31 neurons that individually encoded value for object A (black bars) and on N = 45 neurons that individually encoded view-based value (orange bars).

To investigate how decoding accuracy depended on the number of neurons in the decoding sample in Figure 3Q, we randomly selected a given number of neurons at each step (without replacement) and then determined the percentage correct classification. We repeated this procedure 100 for each tested population size. We performed decoding for randomly shuffled data (shuffled group assignment without replacement) with 1,000 iterations to test whether decoding on real data differed significantly from chance.

For Figure 2K, we used SVM-decoding of value from neuronal activity during the free-viewing (saccade-choice) period. Decoding was performed across all 233 recorded amygdala neurons without pre-selection for value-coding.

For Figure 2O, we used the NN classifier to examine coding across amygdala nuclei. We computed Euclidean distances between single-trial activity vectors and mean activity vectors for different value-levels. We focused on the task period when the first object was presented, as this period likely allowed a ‘pure’ readout of valuation activity irrespective of value comparisons, which required knowledge of the later-occurring second-object reward magnitude. We preselected the 20 neurons with highest value-coding in each nucleus, based on their regression coefficients (Equation 6). We then proceeded as for the decoding analyses described above, except that decoding was not based on Euclidean distances between single-trial vectors but on Euclidean distances between a single-trial test vector and the mean activity vectors for the two alternative groups, calculated from all trials except the test trial. Figure S4F shows the Euclidean distances between low- and high-value groups averaged across the 20 neurons with highest value coefficients in each nucleus.

For Figures 3L, S7I, and S7J, we used SVM-decoding of view-based choice for different groups of trials, organized according to decision difficulty. Decision difficulty was defined as the absolute (unsigned) value difference between the first- and second-viewed choice option; three groups of trials were produced by splitting trials according to terciles of this decision difficulty measure.

For Figure 5C, middle panel, we decoded object choice from trial groups that were separated according to object-viewing sequence (A→B, B→A), thus holding the viewing sequence constant. For Figure 5C, right panel, we trained the classifier to decode object choice from one object-viewing sequence and tested decoding from the alternative viewing sequence (e.g., ‘Train A→B, Test B→A’). For the rightmost bar in Figure 5C, we repeated this cross-decoding procedure but recoded (i.e., inverted) the group labels for the decoding set dependent on the object-viewing sequence. Specifically, when training the decoder on object-viewing sequence A→B, ‘object-A choice’ trials were labeled as ‘group 1’ and ‘object-B choice’ trials were labeled as ‘group 2’; this labeling was reversed for viewing sequence B→A. Thus, based on the viewing sequence the decoder would classify the same input pattern differently. The same approach was used in Figure S14. We note that this analysis illustrates the need and feasibility for an additional processing step in order to readout object choice from the view-based code but it does not indicate how this processing step would be implemented neuronally. Our model suggests expansion recoding as the mechanism to map view-based choice to object-choice, i.e., to explicitly encode view-based choice separately for different viewing sequences, which is supported by data in Figures 5D and 5E.

For Figure 5E, we used a multi-class SVM classifier using the MATLAB function *fitcecoc*. We grouped trials according to the conjunction of object-viewing sequence (A-then-B, or B-then-A) and view-based choice, and then trained the classifier to discriminate the four groups of trials.

##### Representational similarity analysis

We used RSA^17^^,^^77^ to examine how activity across the population of recorded neurons in the amygdala and its subdivisions represented task-related variables as quantified by pairwise correlations between condition-specific neuronal population activity vectors. The RSA approach is a useful analytical tool to examine, for a given task period, which of several task-related variables are encoded particularly strongly at population level, which can differ in principle from the encoding at single-neuron level. For example, although a variable such as view-based choice may be strongly encoded by some individual neurons, this does not necessarily imply strong encoding at population level. Conversely, strong encoding of a given variable at both single-neuron and population level would provide robust evidence that the variable plays an important role in understanding the processing in the studied brain area.

To conduct the RSA analysis, we first calculated, for each recorded neuron, the mean activity related to a specific task event or condition (e.g., activity related to choices for currently viewed and last-viewed objects, activity related to viewing object A or object B, activity related to low and high value levels for object A, etc.). For RSA analyses related to value, we split trials in each session into four equally populated groups (value quartiles). We normalized these condition-specific activities in the same way as for the population decoding analyses described above. For different RSA analyses, we calculated activities in 500-ms fixed time windows (e.g., defined in relation to stimulus presentation) and in 200-ms sliding windows, aligned to a specific task event, that were moved in steps of 20 ms across the trial. Specifically, for the value-based RSA we used 500-ms fixed time windows, collapsed over the first and second object-viewing period. For the choice-based RSA, we used both 500-ms fixed time windows and 200-ms sliding windows, aligned to the onset of the first stimulus and, separately, to target onset. Thus, for a given time window, we calculated the mean activity for given neuron and condition. This procedure generated a condition-by-neuron matrix for a given time window that we then normalized (by removing the mean and dividing by the standard deviation) and used to calculate pairwise Pearson correlation coefficients between conditions across neurons. These matrices of correlation coefficients between conditions are displayed as color-scaled images in Figures 2M, 3K, S4A, S4G, and S8. Row- and column-ordering of conditions was preserved between all RSA matrix displays within a given figure.

To interpret the neuronal RSA matrices and evaluate statistical significance of encoding of particular task-related variables, we generated RSA templates^17^ that captured the representational similarity structure related to specific variables (as described in detail below). For statistical analysis, we performed multiple regression using these templates as regressors to explain a given neuronal RSA matrix. To do so, we concatenated all cells of the neuronal RSA matrix into a vector and regressed this vector on a regressor matrix defined by the concatenated RSA templates.^17^ Statistical significance of coefficients for these RSA regressors was determined using non-parametric permutation tests by shuffling the condition matrix and repeating the regression on the neuronal RSA matrix 10,000 times and then determining the critical t-value corresponding to p < 0.001 across the 10,000 shuffled regressions. Similarly, to test whether a particular neuronal RSA coefficient was significantly larger than another coefficient (Figures 2N, S4B–S4E, and S4H), we computed differences in t-values for these regressors based on the shuffled data and determined a critical t-value difference from the shuffled regressions. We confirmed that the results remained statistically significant when we repeated these analyses using only the unique values from the RSA matrices.

For the value-based RSA (Figure 2M), we defined the following RSA templates: an identity matrix to account for the unity correlation between a condition and itself (diagonal of correlation matrix); an object-specific matrix that took the value of 1 for condition pairs involving the same object and 0 otherwise (this template modeled neuronal responses to particular objects, A or B); a view-based value matrix that modeled four different mean-centered value levels (coded as −2.25, −0.75, 0.75, 2.25) and the pairwise similarity between value levels modeled as the pairwise product of these values, following a previous paper^17^; an object-value matrix defined by the product of the object-identity matrix and the view-based value matrix, thus modeling value similarity only within the same object. (The results from this value-based RSA analysis were robust when we reformulated the value template to treat adjacent value levels as equally similar regardless of their position on the value scale (e.g., the similarity between value levels 1 and 2 is the same as between value levels 2 and 3) and include uniform similarity for equal value levels.^17^ The conditions used to calculate neuronal RSA matrices were defined similarly to these templates, with the four conditions for different value levels resulting from organizing trials according to object-value quartiles. Importantly, when regressing the neuronal RSA matrices on templates, we included both the object-specific value template and the view-based value template (as defined above) as regressors in the same model, so that object-value and view-based value regressors competed to explain variance in the neuronal RSA matrix; the coefficients for these regressors estimated in this way are shown in Figure 2M.

For the choice-based RSA, we defined the following RSA templates: an identity matrix as defined above; an object-specific matrix as defined above; an object-choice matrix that took the value of 1 for condition pairs commonly referring to choice for the same object and 0 for choice for different objects; a view-based choice matrix that took the value of 1 for condition pairs commonly referring to choice for the first-viewed object and 0 for choice for the second-viewed object; a left-choice matrix that took the value of 1 for condition pairs commonly referring to choice for the left-shown object and 0 for choice for the right-shown object.

For Figures 3R and S8D–S8G, we calculated the partial R^2^ for particular variables of the template-based RSA regressions, which indicate the proportion of explained variance of the neuronal RSA matrix that is attributed to a particular variable.

#### Biologically plausible neuronal network model of decision-making

##### Transition from object-based value to view-based value

We built a firing rate computational model, composed of different neural populations, to understand the mechanisms underlying the observed neuronal activity dynamics. We first studied a neural network that has been shown to implement sequential comparisons between stimuli. This network contains neural populations that respond to object values in a state-dependent way, making the response to the second stimulus dependent on the value of the first stimulus.^52^ Specifically, the network is composed of value-comparison (V) neural populations that receive stimulus-dependent input and are coupled to working-memory (M) neural populations that integrate their inputs and send inhibitory feedback to the V neural populations. The firing-rate dynamics of each neural population are given by the following coupled differential equations:

(Equation 12) $$\tau \frac{dV_{i}}{dt}=-V_{i}-w_{VM}f_{V}\left(M_{i}\right)+I_{V,i}\left(t\right)\text{,}$$

(Equation 13) $$\tau \frac{dM_{i}}{dt}=-M_{i}+{w_{MV}f}_{M}\left(V_{i})+w_{MM}{f_{M}(M}_{i}\right)\text{,}$$

where i={1,2} and τ is a time constant. $I_{V,1}$ and $I_{V,2}$ are stimulus-dependent inputs to neural populations $V_{1}$ and $V_{2}$, respectively, taking values equal to $I_{V,1}=1-V_{X}$ and $I_{V,2}=V_{X}$, where $V_{X}$ is the value of the presented object during stimulus presentation (X= A or B; $V_{X}\in \left[0,1\right]$). Thus, $V_{1}$ and $V_{2}$ are negatively and positively tuned to the object’s value, respectively. $f_{V}$ and $f_{M}$ are input-output linear-threshold functions, i.e., $f_{M}\left(u\right)=k{\left[u\right]}_{+}$. The population $M_{i}$ excites itself and inhibits population $V_{i}$ with connection strengths equal to $w_{MM}$ and $w_{VM}$, respectively. $M_{i}$ neural populations are perfect integrators, i.e., $w_{MM}=1$ for k=1 (or $w_{MM}=1/k$, otherwise); they integrate the inputs from neurons $V_{i}$. Thus, after transient activation of $V_{i}$ due to the first stimulus, $M_{i}$ inhibits $V_{i}$ with a strength that is proportional to $I_{V,i}$, even after the stimulus removal. Due to this sustained inhibition, the response of the positively tuned $V_{2}$ neurons to the second stimulus is larger when the value of the second object is larger than the first object’s value (Figure S10B, bottom; see also Figures S13C and S13D). Conversely, the response of the negatively tuned $V_{1}$ neurons to the second stimulus is larger when the value of the second object is lower than the first object’s value (Figure S10B, top; see also Figures S13B and S13D). In conclusion, the responses of $V_{1}$ and $V_{2}$ to the second stimulus are state-dependent, i.e., they depend on the level of inhibition provided by neurons $M_{i}$, which in turn makes them history-dependent. The activity levels of the different neural populations depend on the connectivity between $M_{i}$ and $V_{i}$ (see Figure S13E). A key prediction of this model is reverse tuning during delay activity, an effect that we observed in the data (Figures 5A and 5B). The activation of V neurons provide biased inputs to a decision-making network described in the following.

##### View-based decision-making

We next built a neural network that can implement a decision based on the evidence provided by the V neurons. This network is composed of two coupled neural populations, $C_{1}$ and $C_{2}$, that interact through self-excitation and mutual inhibition. The firing rate of each neural population are given by:

(Equation 14) $$\tau \frac{dC_{1}}{dt}=-C_{1}+\sigma_{1}\left(w_{+}C_{1}-w_{-}C_{2}+{wV}_{1}+I_{0}\right)\text{,}$$

(Equation 15) $$\tau \frac{dC_{2}}{dt}=-C_{2}+\sigma_{2}\left(w_{+}C_{2}-w_{-}C_{1}+{wV}_{2}+I_{0}\right)\text{,}$$

where $w_{+}$ is the strength of the self-excitation, $w_{-}$ is the strength of the mutual inhibition, w scales the inputs from V neurons, and σ is a sigmoid input-output function, $\sigma_{i}\left(u\right)={\left[1+e^{-u/\alpha_{i}}\right]}^{-1}$, with $\alpha_{1}=\alpha_{2}=1$ determining the gain of the sigmoid function. $I_{0}$ represents background input to both neural populations. Depending on this background input: for low $I_{0}$, the network settles into a non-competing stable fixed point; for sufficiently large $I_{0}$, two attractors emerge producing winner-take-all competition between the two neural populations (Figures S11A–S11D). In this last regime, the inputs from V neurons bias the competition toward one of the two attractors. Thus, by controlling the background input, decision-making can be switched on and off.

To switch on the competition between neural populations $C_{1}$ and $C_{2}$ during the presentation of the second stimulus, we modeled the dynamics of $I_{0}$ through a bistable network. This network is composed of excitatory (E) and inhibitory (I) populations, with firing-rate dynamics given by:

(Equation 16) $$\tau \frac{dr_{E}}{dt}=-r_{E}+\sigma_{E}\left(w_{EE}r_{E}-w_{EI}r_{I}+I_{E}+w_{F}b\right)\text{,}$$

(Equation 17) $$\tau \frac{dr_{I}}{dt}=-r_{I}+\sigma_{I}\left(w_{IE}r_{E}-w_{II}r_{I}+I_{I}\right)\text{,}$$

(Equation 18) $$\tau_{F}\frac{dw_{F}}{dt}=k_{1}\left(1-w_{F}\right)+k_{2}r_{E}w_{F}\text{,}$$

where $w_{XY}$ represent the connection strength from population Y to population X, and $I_{E}$ and $I_{I}$ are constant inputs to E and I neurons, respectively. To produce bistability, the input-output function of the I population has a lower gain than the one of the *E* population: $\alpha_{E}=1$ and $\alpha_{I}=3$. Depending on the input b to the E population, the network transits from a low-activity state to a high-activity state (Figures S11E–S11H). The decision-making module described above is switched on by setting $I_{0}\left(t\right)=r_{E}\left(t\right)$. Short-term synaptic facilitation^78^ modulates the strength ($w_{F}$) of the input b (Equation 17) and ensures that decision-making is switched on during the presentation of the second stimulus. The constant $k_{1}/\tau_{F}$ is the rate of synaptic recovery and the term $k_{2}r_{E}w_{F}$ represents multiplicative synaptic facilitation. The input b corresponds to the activity of object-selective neurons (Figure S12A, left), which firing-rates are noted neurons $r_{A}$ and $r_{B}$, i.e., $b=w_{b}\left(r_{A}+r_{B}\right)$.

In classical attractor models of decision-making, this ‘switch’ is modeled as an external input to both competing neural populations, representing a modulation from a different brain area that is not explicitly modeled.^5^^,^^79^^,^^80^ Here, instead of an external input, we used the above bistable network that accumulates the activity of other neural population of the model and automatically triggers competition. However, replacing the bistable network by an external input would not change the results. We note that our aim was not to present a model that provides a strong quantitative fit to neurophysiological data, as the field still lacks much critical data regarding primate amygdala neurons. For example, the density of recurrent collaterals in different subnuclei remains an important open question that would affect the efficacy of the decision and memory neurons.

We further note that we focused our analyses on the model’s key computations that explained the view-based decision process and translation to object-choice signals. We do not suggest that all model components are necessarily directly implemented in local amygdala circuits; for example, the memory and switch mechanisms constitute ‘ancillary’ processes that may involve inputs from other structures. We further note that the particular strength of value coding during the memory period is not a critical model prediction, as this varies with the coupling strength between the M and V. The crucial parameter to sustain memory is the self-coupling of population M, which allows the feedback inhibition to the V population to depend on the first stimulus to implement value-comparison between sequentially viewed stimuli (Figure S13). Thus, model parameters can be chosen to change the activation level of M populations while retaining the network’s ability to compare sequential stimuli.

##### Object-sequence neurons

We found neurons in the amygdala that combined information about object identity and viewing sequence. These neurons have larger activation during the second stimulus' presentation if object A was presented first (A 1st neuron type, A_1_) or if object A was presented second (A 2nd neuron type, A_2_; Figure S12A, right). Note that "A 2nd neurons" could be also called "B 1st neurons". We modeled the dynamics of A_1_ and B_1_ neurons using short-term synaptic depression.^78^ The neurons receive inputs from object-selective neurons. The dynamics are given by:

(Equation 19) $$\tau \frac{dr_{A}}{dt}=-r_{A}+I_{A}\text{,}$$

(Equation 20) $$\tau \frac{dr_{B}}{dt}=-r_{B}+I_{B}\text{,}$$

(Equation 21) $$\tau \frac{dA_{1}}{dt}=-A_{1}+{w_{A}r}_{A}+r_{B}\text{,}$$

(Equation 22) $$\tau \frac{dB_{1}}{dt}=-B_{1}+r_{A}+{w_{B}r}_{B}\text{,}$$

(Equation 23) $$\tau_{s}\frac{dw_{A}}{dt}=k_{1}\left(1-w_{A}\right)-k_{3}A_{1}w_{A}\text{,}$$

(Equation 24) $$\tau_{s}\frac{dw_{B}}{dt}=k_{1}\left(1-w_{B}\right)-k_{3}B_{1}w_{B}\text{,}$$

where $I_{A}$ and $I_{B}$ are inputs signaling objects A and B (i.e., $I_{X}=1$ during presentation of stimulus X and $I_{X}=0$ otherwise), respectively; $k_{1}/\tau_{s}$ is the rate of synaptic recovery and the terms ${-k}_{3}A_{1}w_{A}$ and ${-k}_{3}Bw_{B}$ represent depression of synapses $w_{A}$ and $w_{B}$, respectively. Symmetric equations can be written for neuron types A_2_ and B_2_. We choose the synaptic time constant $\tau_{s}$ to be slow, so that if object B was presented first, the synapse $w_{A}$ would be depressed by the arrival of the second stimulus (A), thus reducing the response of A_1_ during the second stimulus. Conversely, if object A was presented first, the synapse $w_{A}$ would not be affected by the second stimulus (B), thus the response of A_1_ would be larger than in the previous case. The dynamics of A_1_, A_2_, B_1_, and B_2_ neurons are presented in Figure S10. The activities of object-sequence neurons are inputs to combination neurons that we describe below.

##### Expansion recoding and object-based decision-making

Four types of combination neurons combine inputs from object-sequence neurons (A_1_, A_2_, B_1_, and B_2_ neurons) and view-based choice neurons (C_1_ and C_2_ neurons) to signal object choice for specific object-viewing sequences. We found experimental evidence of this type of neurons, especially in the BL (Figures 5D and S14). The dynamics of combination neurons are given as:

(Equation 25) $$\tau \frac{dC_{A1}}{dt}=-C_{A1}+f\left(A_{1}+C_{1}\right)\text{,}$$

(Equation 26) $$\tau \frac{dC_{A2}}{dt}=-C_{A2}+f\left(A_{2}+C_{2}\right)\text{,}$$

(Equation 27) $$\tau \frac{dC_{B1}}{dt}=-C_{B1}+f\left(B_{1}+C_{1}\right)\text{,}$$

(Equation 28) $$\tau \frac{dC_{B2}}{dt}=-C_{B2}+f\left(B_{2}+C_{2}\right)\text{,}$$

where the input-output function f is a linear-threshold function. Finally, the sums $s_{A}=C_{A1}+C_{A2}$ and $s_{B}=C_{B1}+C_{B2}$ provide inputs to a winner-take-all network that chooses the object with higher value:

(Equation 29) $$\tau \frac{dC_{A}}{dt}=-C_{A}+\sigma_{1}\left(w_{+}C_{A}-w_{-}C_{B}+{w_{c}s}_{A}+I_{1}\right)\text{,}$$

(Equation 30) $$\tau \frac{dC_{B}}{dt}=-C_{B}+\sigma_{2}\left(w_{+}C_{B}-w_{-}C_{A}+w_{c}s_{B}+I_{1}\right)\text{,}$$

where $I_{1}$ is a constant background input. Neurons $C_{A}$ and $C_{B}$ explicitly signal the choice for object A or object B, irrespective of viewing sequence. We simulated the full model in the presence of additive uncorrelated Gaussian noise (with amplitude η) injected to Equations 12, 13, 14, 15, 16, 17, 19, 20, 21, 22, 25, 26, 27, 28, 29, and 30.

Model parameters: $w_{MM}=1$, $w_{MV}=w_{VM}=0.6$; $w_{+}=2.5$, $w_{-}=2$, w=0.1; $w_{EE}=16$, $w_{EI}=9$, $w_{IE}=10$, $w_{II}=7$, $w_{b}=0.2$; $w_{c}=0.5$; $I_{E}=-3$, $I_{I}=-1$; $I_{1}=-1.6$; $k_{1}=0.015$, $k_{2}=1.1$, $k_{3}=0.22$; $f_{V}\left(u\right)={\left[u\right]}_{+}$, $f_{M}\left(u\right)=0.2\times {\left[u\right]}_{+}$, $f\left(u\right)={\left[u-1.35\right]}_{+}$, $\sigma_{1}\left(u\right)=\sigma_{2}\left(u\right)=\sigma_{E}\left(u\right)={\left[1+e^{-u}\right]}^{-1}$, $\sigma_{I}\left(u\right)={\left[1+e^{-u/3}\right]}^{-1}$; τ=10 ms, $\tau_{F}=500$ ms, $\tau_{s}=1$ s; η=0.025. We note that we make no strong assumptions about how these model parameters are set up initially. Here, we tuned these parameters to match the key amygdala neuron types recorded in the experiment and to examine their signal dynamics. We explore in the Discussion how the described circuits may emerge in self-organizing networks, without precise tuning or fixed-point dynamics.

##### Consideration of alternative model architectures

We explored variations to the architecture of the computational model shown in Figure 4B as briefly summarized here.

First, we considered the classical attractor-based decision circuit operating with mutual inhibition and recurrent excitation.^5^ As this classical model does not contain a mechanism to bridge the delay between sequentially applied stimuli, we applied the stimuli simultaneously. As expected, the model successfully selected the option with the higher value input; however, because each population of object-specific value neurons projected only to one population of decision neurons (e.g., V_A_→C_A_ and V_B_→C_B_), the model could not solve decision-making for additional objects without introducing additional object-specific decision circuits (e.g., V_A_→C_A_ and V_c_→C_C_).

Second, we considered model variations without the switch module. Depending on the background input $I_{1}$ to view-based decision neurons (C_1_, C_2_), the model without switch module would either engage in continual, premature decision-making when the value input of the first option was applied, without settling into a stable state, or it would not engage in any decision-making due the lack of excitatory drive (Figure S11I). Thus, the proposed switch mechanism is useful in enabling the network to start the decision computation only once all choice options have been viewed and to prevent premature decision-making.

Third, we considered models without abstract view-based neurons. To do so, we removed the cross-connections from object-value neurons to view-based neurons (cf. Figure 4B), so that V_A_ would project to V_1_ but not V_2_, and vice versa for V_B_. Removing these connections effectively turned the abstract view-based neurons into object-specific neurons. We found that this network always selected the second option, as each population of view-based neurons now only responded to one object (A or B, shown first or second), which removed the integral-feedback control mechanism and thus responses to the second object no longer depended on the value of the first object. As a consequence, the value signal of the second option always out-competed the memory trace of the value of the first option.

Fourth, to allow for fair competition between first and second object, we modified the model without abstract view-based neurons by making both V_1_ and V_2_ positively tuned to value, by giving a positive sign to the synaptic weight W_MC_ from memory neurons M_1_ and M_2_ to V_1_ and V_2_, and by setting the weights W_MC_ to a relatively weak value of 0.8. Similar to our main model, this model variant correctly selected between the competing, sequentially viewed first and second object and produced choice signals that depended on the absolute value difference between objects. However, as with the classical attractor-based decision circuit for simultaneously applied options (see our first point above), it could not solve decision-making for additional objects without introducing an additional object-specific decision circuit. Thus, abstract view-based neurons serve the useful function of enabling a decision circuit to select flexibly among varying pairs of choice objects (solving the ‘many-objects problem’).

## Data Availability

• Data reported in this paper will be shared by the lead contact upon request. • All original code has been deposited at GitHub: https://github.com/FGrabenhorst/Grabenhorst_AmygdalaChoice_Neuron2023/releases/tag/v1.0 (https://doi.org/10.5281/zenodo.8268856) and is publicly available as of the date of publication. • Any additional information required to reanalyze the data reported in this work paper is available from the lead contact upon request

## Supplemental information

Document S1. Figures S1–S14 and Tables S1 and S2

Document S2. Article plus supplemental information
