# Supplementary material for: A view-based decision mechanism for rewards in the primate amygdala
Source: Neuron. 2023 Dec 6;111(23):3871–3884.e14. doi: 10.1016/j.neuron.2023.08.024 (PMC10914681; doi:10.1016/j.neuron.2023.08.024)
Supplement: Document S1. Figures S1–S14 and Tables S1 and S2 [file mmc1.pdf]

Neuron, Volume *111*

Supplemental information

A view-based decision mechanism

for rewards in the primate amygdala

Fabian Grabenhorst, Adrián Ponce-Alvarez, Alexandra Battaglia-Mayer, Gustavo Deco, and Wolfram Schultz

SUPPLEMENTAL INFORMATION

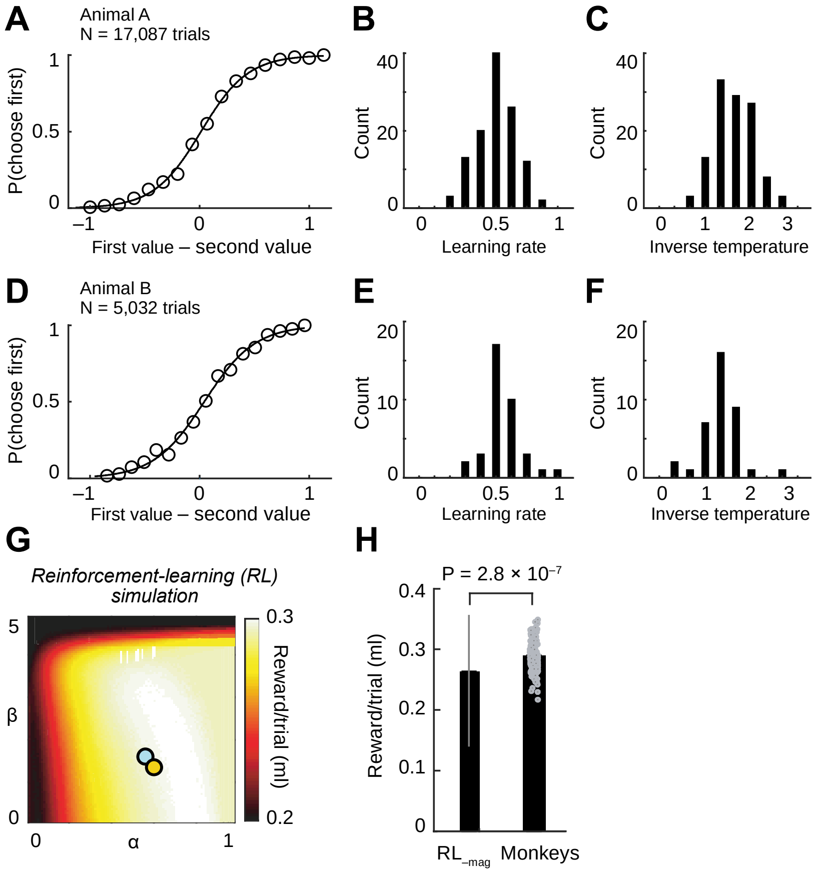

Fig. S1. Reinforcement learning in both animals. Related to Fig. 1. **(A)** Psychometric curve linking value difference between first- and second-viewed option in animal A, derived from best-fitting RL model (Eq. 1-5), to the choice probability for the first option. **(B)** Histogram of learning rate parameter across sessions in animal A for best-fitting RL model. **(C)** Histogram of inverse temperature parameter across sessions in animal A. **(E-F)** Results for animal B. **(G)** Monkeys' choices approximate optimal reward maximization. Obtained reward per trial as a function of RL model parameters from simulations and monkeys' choices (blue/orange points: monkey A/B). **(H)** Across sessions, monkeys outperform alternative RL models that ignore transiently cued magnitudes, supporting the notion that the monkeys used the transiently cued magnitude information for decision-making.

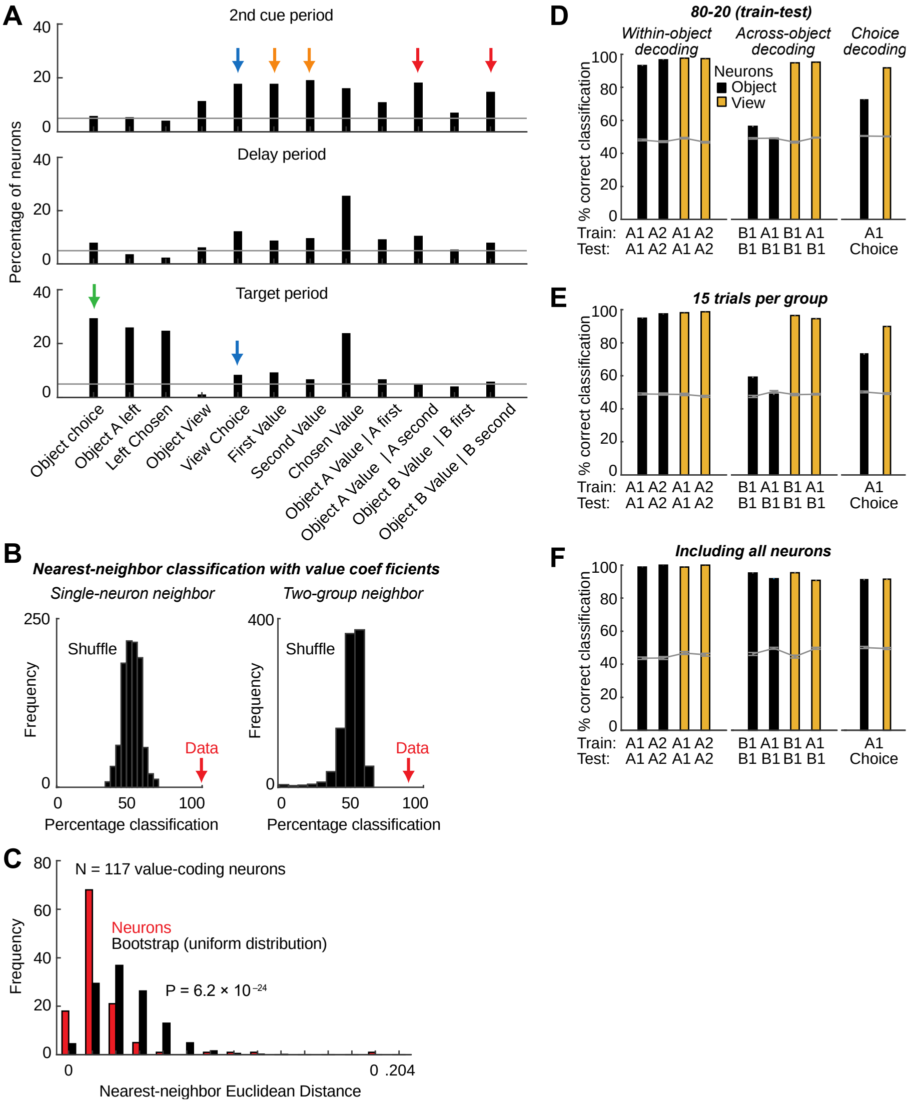

Fig. S2. Stepwise regression on neuronal responses and decoding robustness tests. Related to Fig. 2. **(A)** Percentage of neurons encoding specific task variables, identified with stepwise regression in three task periods. All variables were included in the starting set for the stepwise selection procedure. These results support several of the main findings. First, when the second cue was being viewed (top), both view-based values ('First value', 'Second value', indicated by orange arrows) and object values ('Object A value | A second', 'Object B value | B second', indicated by red arrows) were encoded in the population of amygdala neurons. Second, view-based choice ('View choice', blue arrow) was encoded early, during the second cue period (top) and preceded the encoding of object-based choice ('Object choice', green arrow), which was primarily encoded in the target period (bottom). **(B)** We tested whether the value regression coefficients shown in Fig 2G provided evidence for clustering into two groups corresponding to object-value and view-based value neurons. We performed nearest-neighbor classification on the angles in the space of value coefficients. We were primarily interested in whether, for a given neuron, this angle would fall onto either of the two axes corresponding to object-specific value coding (object A or object B) or whether the angle would lie on the diagonal between these axes, corresponding to view-based value coding. We therefore sign-corrected the coefficients (i.e., ignoring whether a given neuron would code value for object A or object B) resulting in angles between 0 and 45 degrees. For each value-coding neuron (N = 117), we calculated the Euclidean Distance between its value coefficients and those of the remaining neurons (leave-one-out cross-validation) to classify the tested neuron as either object-based value coding or view-based value coding, depending to which group the Euclidean Distance was smallest. Classification based on assignment to the closest 'single-neuron neighbor' resulted in 116 correct classifications (99% correct), classification based on

the closest ‘two-groups neighbor’ (the mean coefficients from object-value and view-based value neurons) resulted in 105 correct classifications (89% correct). For comparison, Euclidean Distance to an overall mean of value coefficients was smaller than the distance to the mean of either object- and view-based value groups in only 26 cases (22%). **(C)** Distribution of Euclidean Distances from the nearest-neighbor approach in (B) compared to the Euclidean Distances obtained from a uniform bootstrap distribution (significance obtained from Wilcoxon test). **(D, E)** Robustness tests. The results in Fig. 2E were replicated when we varied the parameters of the decoding method (i.e., using 80% of data for decoder training and 20% for decoder testing, (D), and increasing the inclusion criterion to 15 trials per decoding group (E)). **(F)** Results for the decoding analyses in Fig. 2E when all recorded amygdala neurons were included in the analyses.

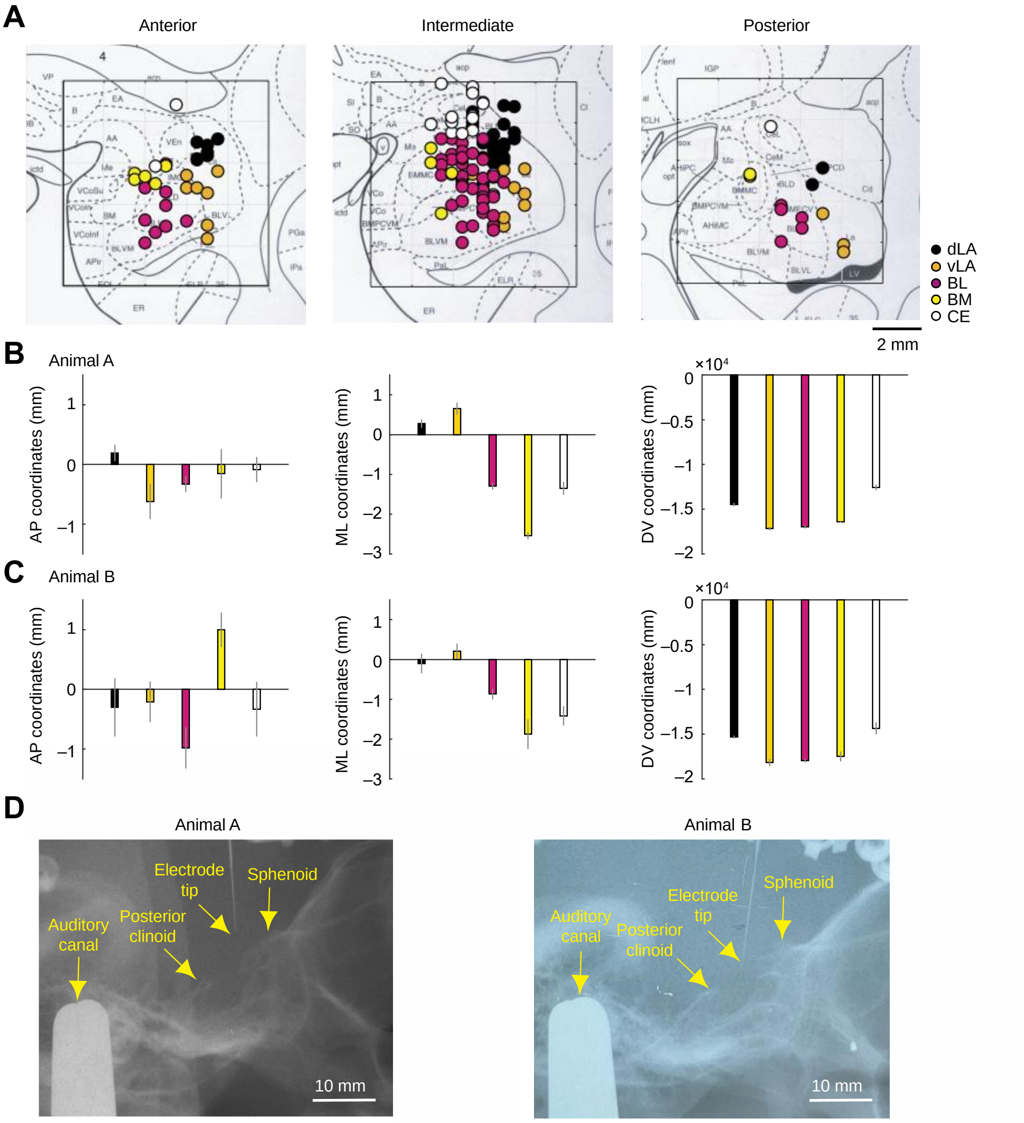

E

	Amygdala nucleus					
	LA	dLA	vLA	BL	BM	Ce
Animal A	76 (51)	47 (31)	29 (20)	68 (43)	13 (8)	23 (16)
Animal B	17 (13)	5 (3)	12 (10)	26 (20)	4 (3)	6 (6)
Combined	93 (64)	52 (34)	41 (30)	94 (63)	17 (11)	29 (22)

Fig. S3. Overview of recording sites in amygdala nuclei. Related to Fig. 2. **(A)** Recording positions were determined using histological reconstructions based on electrolytic lesions and marker pins, and stereotaxically referenced coordinates for recordings for each neuron. Reconstructed recording positions were overlaid onto a stereotaxic atlas[71] of the macaque brain at different anterior-posterior levels and neurons were assigned to different amygdala nuclei. **(B, C)** Mean coordinates of recording positions (± s.e.m) for recorded neurons in anterior-posterior (AP), medio-lateral (ML) and dorsoventral (DV) dimensions for animal A and animal B. Coordinates are shown separately for different amygdala nuclei; color code as in panel (A). Coordinates are referenced to recording grid system. **(D)** Lateral view X-rays for animal A and animal B showing recording electrode in relation to bone landmarks and auditory canal indicating the position of the amygdala posterior and ventral to the anterior sphenoid bone and anterior to the posterior clinoid process. **(E)** Number of neurons recorded in different amygdala subnuclei. Numbers in parentheses indicate number of recording sessions.

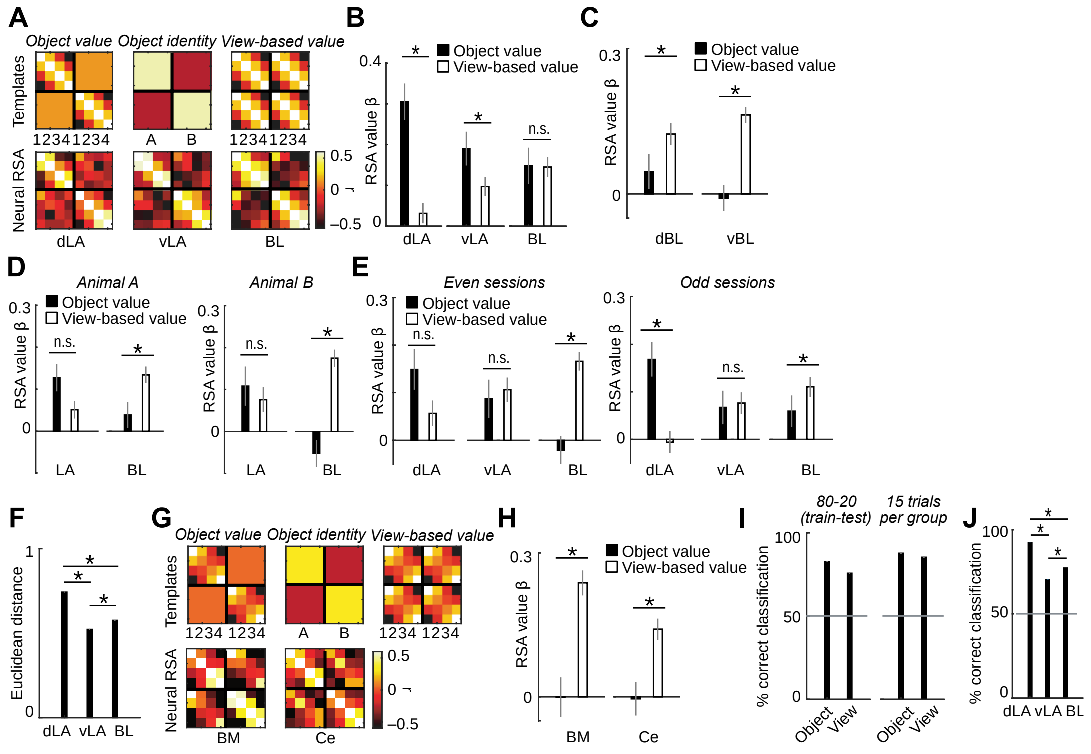

Fig. S4. Representational Similarity Analysis: alternative value template and results in additional amygdala nuclei. Related to Fig. 2. **(A)** An alternative formulation of the RSA value templates (top) treats adjacent value levels as equally similar regardless of their position on the value scale (e.g., the similarity between value levels 1 and 2 is the same as between value levels 2 and 3) and includes uniform similarity for equal value levels (matrix diagonal[17]). Neuronal RSA matrices (bottom) are the same as in Fig. 2M. In dLA, only the object-based regressor was significant ($P < 0.0001$); in vLA and BL, both object- and view-based regressors were significant ($P < 0.0001$). **(B)** Multiple regression of neuronal RSA on these alternative value templates confirms our main result of a transition from a predominantly object-value code to a view-based value code across amygdala nuclei (significance based on bootstrap; $P < 0.005$). **(C)** RSA control analysis. Different from the dorso-ventral gradient observed in lateral nucleus (Fig. 2N), a gradient for object-value and view-based value coding was not found in BL (indicated by similar emphasis on view-based value coding in both dBL and vBL; significance based on bootstrap; *: $P < 0.005$). **(D)** Multiple regression of neuronal RSA on templates, separately within each animal. For this analysis, we collapsed data from dLA and vLA into LA. **(E)** Multiple regression of neuronal RSA on templates, separately for data subsets split into odd and even sessions (neuron numbers for odd/even sessions: dLA: 25/27; vLA: 21/20; BL: 48/46). **(F)** Euclidean distances between population vectors for high vs. low value levels within a given object. dLA showed the clearest separation of value levels within objects. **(G, H)** RSA analysis for amygdala nuclei downstream of BL. Similar to BL, population-activity patterns in BM and Ce reflected view-based rather than object-based values (significance based on permutation tests; *: $P < 0.005$). **(I, J)** Robustness tests. Results from Fig. 2K and Fig. 2O were replicated when we varied the parameters of the decoding method (i.e., using 80% of data for decoder training and 20% for decoder testing, and increasing the inclusion criterion to 15 trials per decoding group).

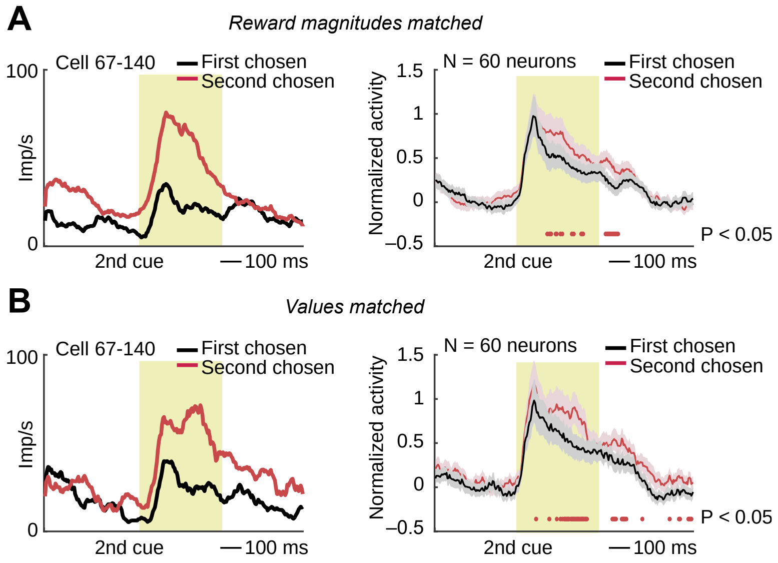

Fig. S5. Control analyses for view-based choice signals with matched reward magnitudes and matched value levels. Related to Fig. 3. **(A)** Activity of the example neuron from Fig. 3A (left) and population activity of view-based choice neurons (right; mean ± s.e.m.) on trials in which the cued reward magnitudes for the first- and second-viewed options were identical. The neurons reflected the animals' trial-specific choice for the first- or second-viewed option even when reward magnitudes were identical, confirming that view-based choice signals were not explained by reward magnitude. **(B)** Activity of the example neuron from Fig. 3A (left) and population activity of view-based choice neurons (right; mean ± s.e.m.) on trials in which the value for the first- and second-viewed options were closely matched (value-matching was performed by selecting trials that corresponded to the lowest decile of absolute value difference in a given session). The neurons reflected the animals' trial-specific choice for the first- or second-viewed option even when values were matched, confirming that view-based choice signals were not explained by value.

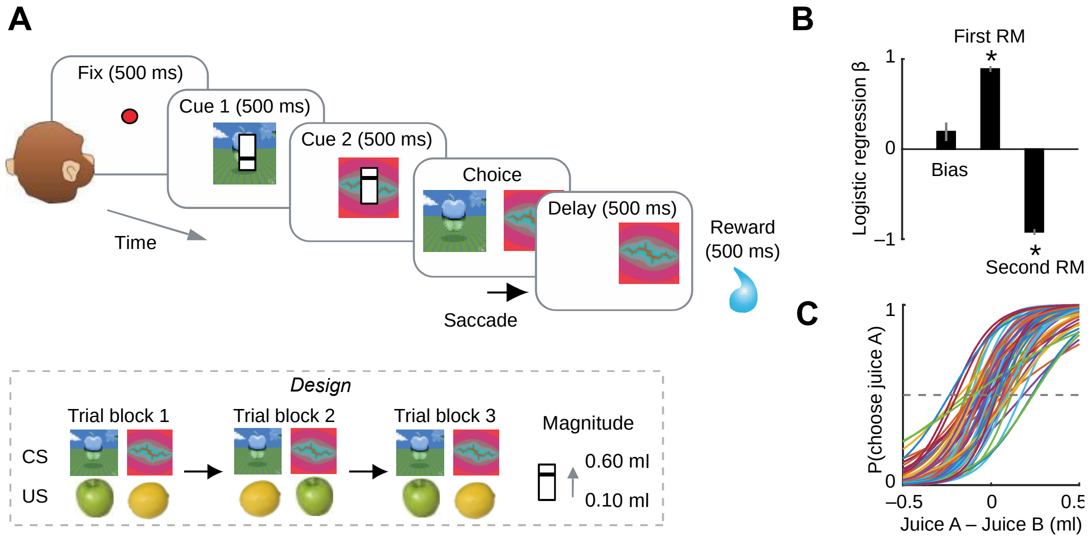

Fig. S6. Control task with different reward types. Related to Fig. 3. **(A)** As in the main task, the monkey fixated sequentially presented objects composed of a picture, which in this task predicted a specific juice reward, and a bar stimulus indicating reward magnitude. Inset: Task design within a session. The association between a particular picture (conditioned stimulus, CS) and a particular juice (unconditioned stimulus, US) typically changed twice in a recording session to distinguish neuronal object-coding from reward-coding. **(B)** Logistic regression of choice for the first-viewed option on first and second reward magnitudes (N = 12,325 trials). **(C)** Psychometric curves in all testing sessions linking choice probability for juice type A to the difference in reward magnitude between juice A and juice B (specific juice types varied across sessions). Variability of indifference points (intersections between curves and hatched line) around the point of objective magnitude equivalence (position zero on x-axis) indicates that the different reward types were behaviorally meaningful and elicited subjective values and preferences.

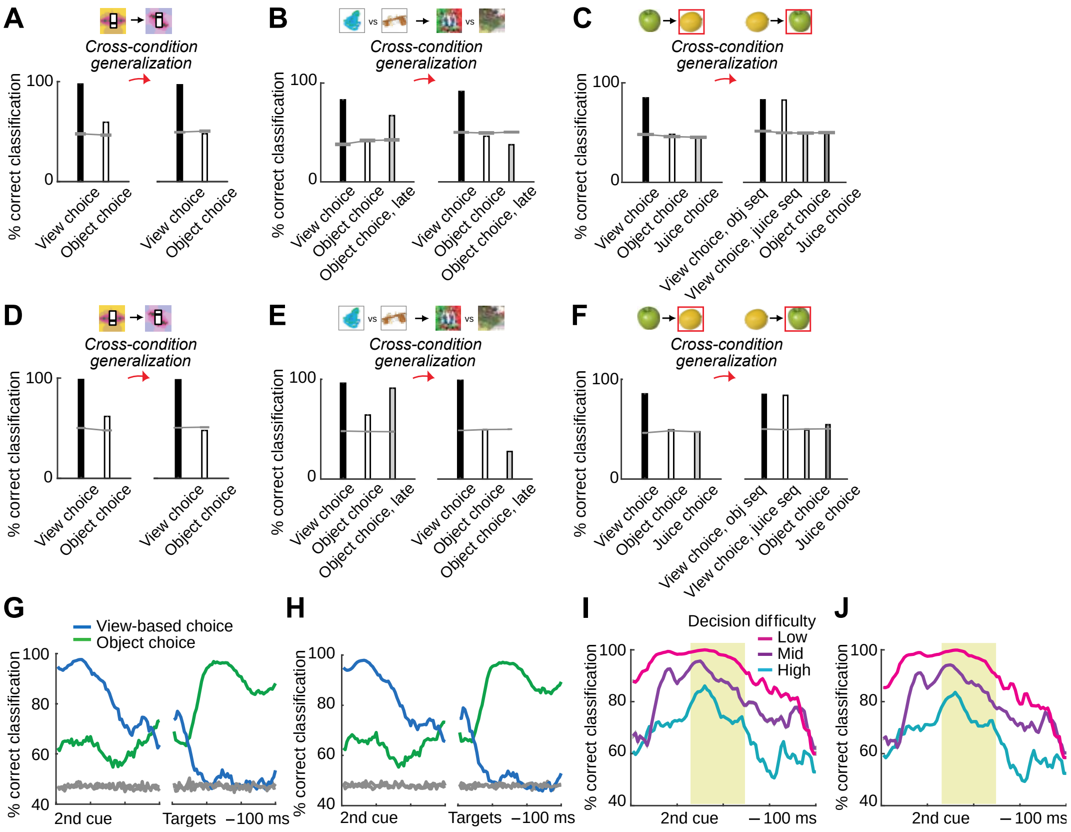

Fig. S7. Cross-condition generalization of view-based choice signals in three tasks, and robustness tests. Related to Fig. 3. Results of population decoding show that view-based choice signals generalize across object-viewing sequence (A), different visual object sets (B) and different reward types (C). **(A)** Main task: a linear SVM-classifier was trained to decode choice from amygdala population activities. Left: training and testing were performed on the same conditions (i.e., trial types), with cross-validation. Right: training was performed using data from one object-viewing sequence (e.g., A-then-B) and classification was tested using the alternative object-viewing sequence (B-then-A). Black/white: classification of view-based/object-based choice. **(B)** Control task with four visual objects. Left: training and testing were performed on the same conditions, with cross-validation. Right: training was performed using data from one object set (e.g., A, B) and classification was tested using the alternative object set (C, D). Black/white/grey: classification of view-based choice during second cue/object-based choice during second cue/object-based choice during late (target) period. **(C)** Control task with different reward types. Left: training and testing were performed on the same conditions, with cross-validation. Right, 'View choice, obj seq': training was performed using data from one visual object sequence (e.g., A-then-B) and classification was tested using the alternative object sequence (B-then-A). Note that object-reward associations changed throughout the session. Right, 'View choice, juice seq': training was performed using data from one reward-viewing sequence (e.g., lemon-then-apple) and classification was tested using the alternative reward sequence (apple-then-lemon). Black/white/grey: classification of view-based/object-based/juice-based choice. **(D-F)** Robustness tests. The results in (A-C) were replicated when we varied the parameters of the decoding method (increasing the inclusion criterion to 15 trials per decoding group). **(G, H)** Robustness tests. The results in Fig. 3J were replicated when we varied the parameters of the decoding method (i.e., using 80% of data for decoder training and 20% for decoder testing, (G), and increasing the inclusion criterion to 15 trials per decoding group (H)). **(I, J)** Robustness tests. The results in Fig. 3L were replicated when we varied the parameters of the decoding method (i.e., using 80% of data for decoder training and 20% for decoder testing, (I), and increasing the inclusion criterion to 15 trials per decoding group (J)).

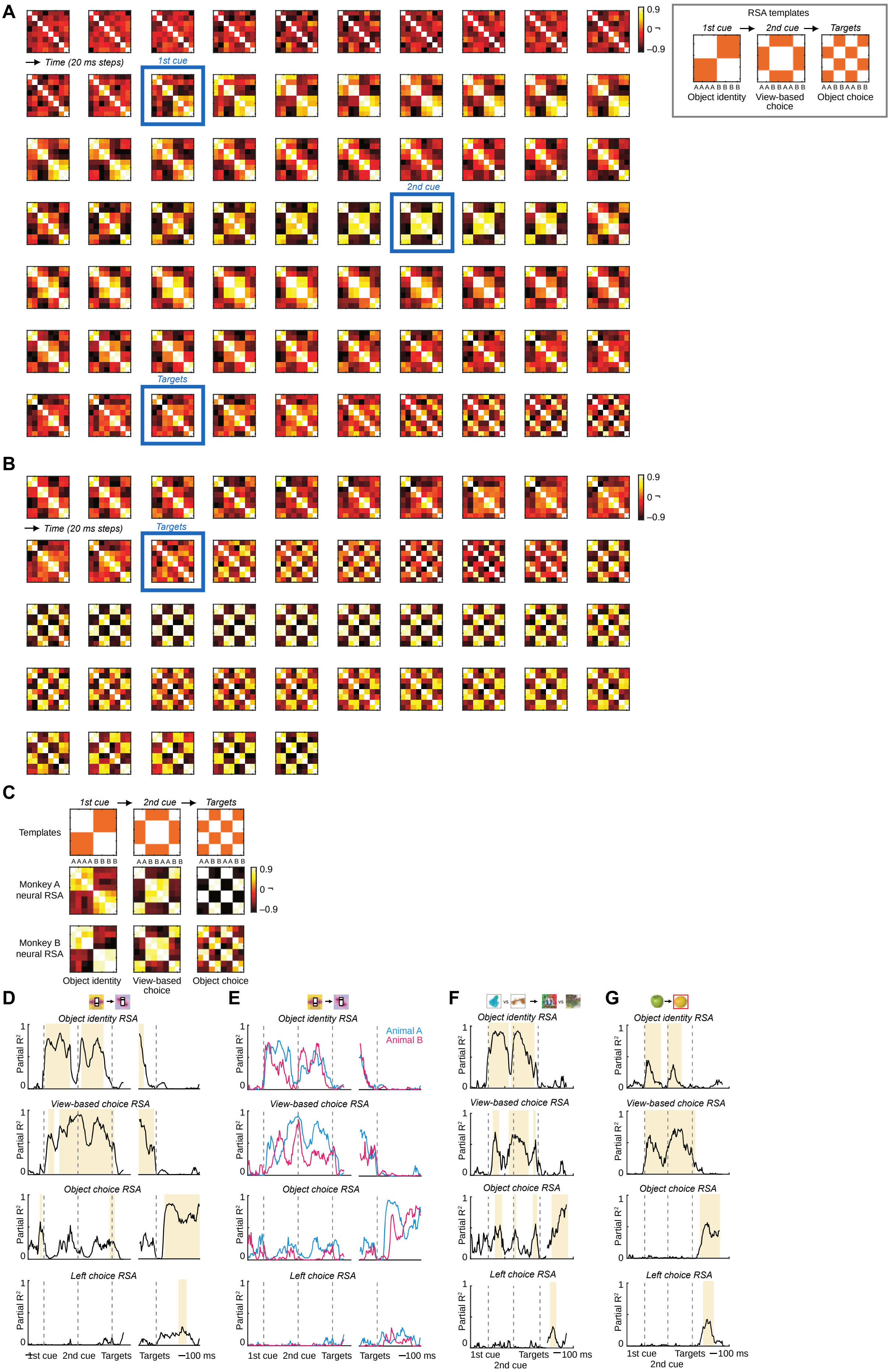
A
Time (20 ms steps)
1st cue
2nd cue
Targets
0.9
0
–0.9
RSA templates
1st cue
2nd cue
Targets
AAAABBBB
Object identity
AABBAABB
View-based choice
AABBAABB
Object choice
B
Time (20 ms steps)
Targets
C
Templates
Monkey A neural RSA
Monkey B neural RSA
D
E
F
G
Object identity RSA
View-based choice RSA
Object choice RSA
Left choice RSA
Partial R²
Animal A
Animal B
1st cue
2nd cue
Targets
100 ms

Fig. S8. Dynamics of RSA patterns across trial periods, animals and tasks. Related to Fig. 3. Neuronal RSA matrices were calculated in 200-ms time windows moved in 20-ms steps. Event onsets shown in blue rectangles. Top: RSA templates. **(A)** RSA calculated for activity aligned to first-cue onset. **(B)** RSA calculated for activity aligned to target onset. The figure shows that population activity patterns transiently reflected an abstract, view-based choice signal during the second-cue period, before transitioning to an object-choice pattern during the target period. **(C)** RSA patterns in the main task shown separately for both animals. The transition from coding of object identity to view-based choice to object choice was robust across animals. **(D-F)** Sliding-window regression of population activity (representational-similarity structure) on RSA templates across the three tasks. Shown are the coefficients of partial determination, derived from the multiple regression model, for different variables. Yellow shaded areas indicate periods of significant RSA regression ($P < 0.005$, determined from bootstrap). **(D)** RSA regression in main task. **(E)** RSA regression in main task showing similar effects in both animals. **(F)** RSA regression in four-objects control task. **(G)** RSA regression in two-juices control task. The figure shows that the dynamics of population coding were similar across tasks. Specifically, a view-based choice code appeared transiently at the time of the second cue and preceded an object-based choice code.

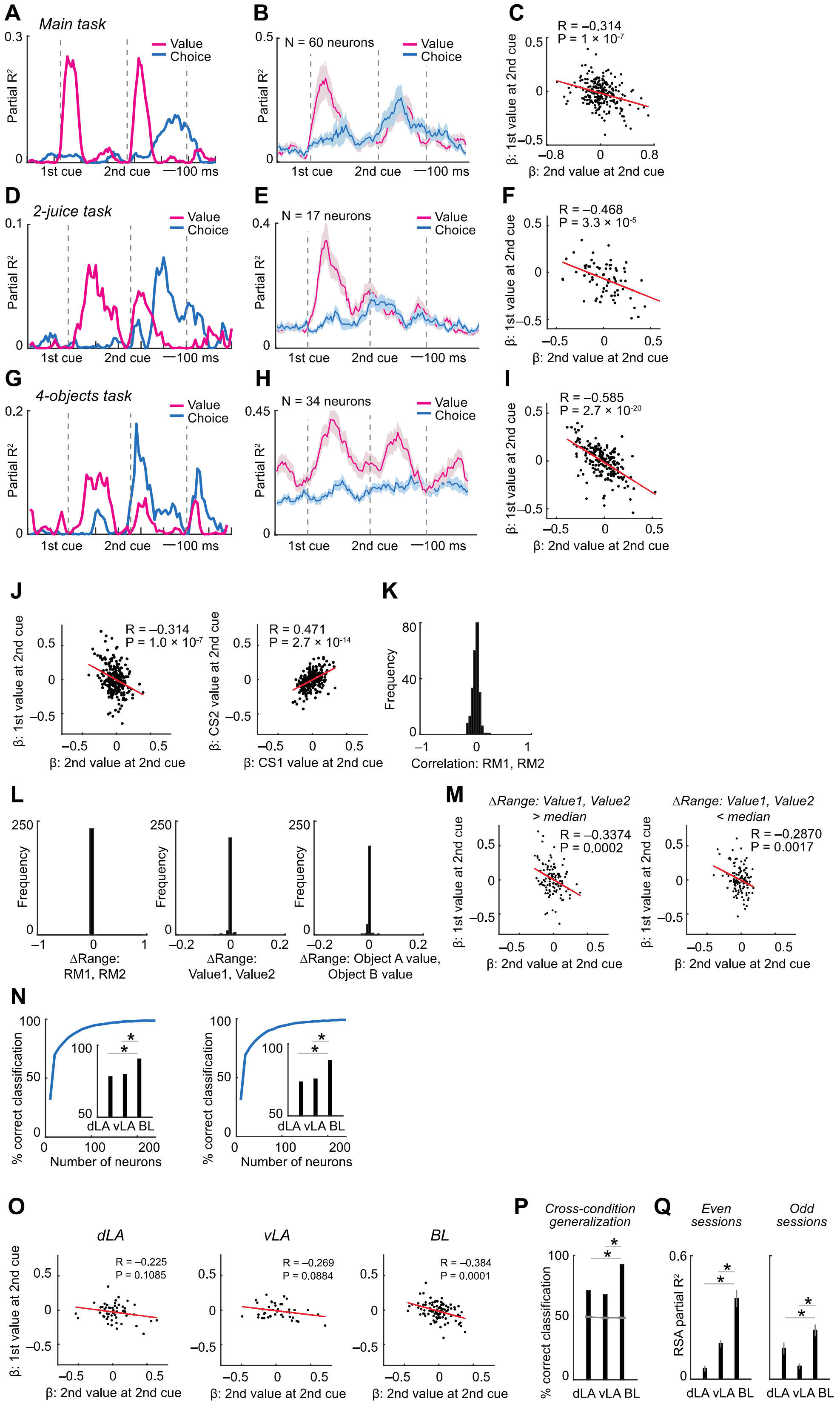

Fig. S9. Neuronal decision signatures and controls across tasks and nuclei. Related to Fig. 3.
(A) Neuronal signatures of decision computations in amygdala across three tasks. (A) Single amygdala neurons encoding a value-to-choice transition in the main choice task. The neuron encoded view-based value at the first and second cue before encoding the monkey's view-based choice. **(B)** Value-to-choice transition encoded by a population of amygdala neurons (N = 60; mean ± s.e.m.). The

neurons were selected for showing a significant view-based choice regressor in the second-cue period. Although not pre-selected for value coding, the neuronal population showed a clear value signal preceding the choice signal. **(C)** Signature of value comparison. Anti-correlated neuronal value coefficients between first and second cue (N = 233 neurons). **(D)** Single-neuron value-to-choice transition in the two-juices task. **(E)** Population value-to-choice transition in the two-juices task. Neurons selected for encoding view-based choice (N = 17). **(F)** Anti-correlated neuronal value coefficients between first and second cue in the two-juices task (N = 72 neurons). **(G)** Single-neuron value-to-choice transition in the four-objects task. **(H)** Population value-to-choice transition in the four-objects task. Neurons selected for encoding view-based choice (N = 34). **(I)** Anti-correlated neuronal value coefficients between first and second cue in the four-objects task (N = 205 neurons).
(J-N) Control analysis for anti-correlated neuronal value coefficients for first- and second-viewed choice options, and decoding robustness tests. (J) Anti-correlation was observed for view-based options (defined for first- and second-viewed option) but not object-based options (defined for different conditioned stimuli, i.e., objects: CS1 and CS2). Value coefficients for different objects in the second cue period were positively correlated, indicating view-based value processing with a common encoding scheme for currently viewed object, rather than object-based value comparison. **(K)** Reward magnitudes, which provided the basis calculating value-regression coefficients for these analyses, were not correlated between first and second option. Histogram of correlation coefficients calculated between first and second reward magnitudes across testing sessions. **(L)** Control for differences in value range. Value ranges were equal between first and second option, shown for reward magnitudes (left, these constituted the basis for the anti-correlation analyses between neuronal value coefficients), integrated values from reinforcement learning model (middle) and object values (right). **(M)** Further control for value range. Similar anti-correlation between neuronal value-slopes for first and second option was found even when median-splitting data based on small range variation shown in panel (C). **(N)** Decoding robustness test for results shown in Fig. 3Q. Results from Fig. 3Q were replicated when we varied the parameters of the decoding method (i.e., using 80% of data for decoder training and 20% for decoder testing, left, and increasing the inclusion criterion to 15 trials per decoding group, right. Decoding view-based choice for different decoding sample sizes (mean ± s.e.m.). Insets: decoding from specific nuclei (N = 20 neurons per nucleus; * $P < 0.001$, Wilcoxon test).
(O-Q) Signatures of decision computation across amygdala nuclei. (O) Anti-correlation of neuronal value slopes was only significant in BL. **(P)** Signatures of abstract representation defined by the cross-condition classification performance for decoding view-based choice were strongest in BL. Results obtained when training an SVM-classifier to decode view-based choice from one object-viewing sequence (e.g., A-then-B) and testing on the alternative sequence (B-then-A). *: $P < 0.001$ (Wilcoxon test). **(Q)** Neuronal RSA regression of view-based choice in different nuclei performed separately for subsets of data split into even and odd sessions. (*: $P < 0.005$, significance determined by permutation test).

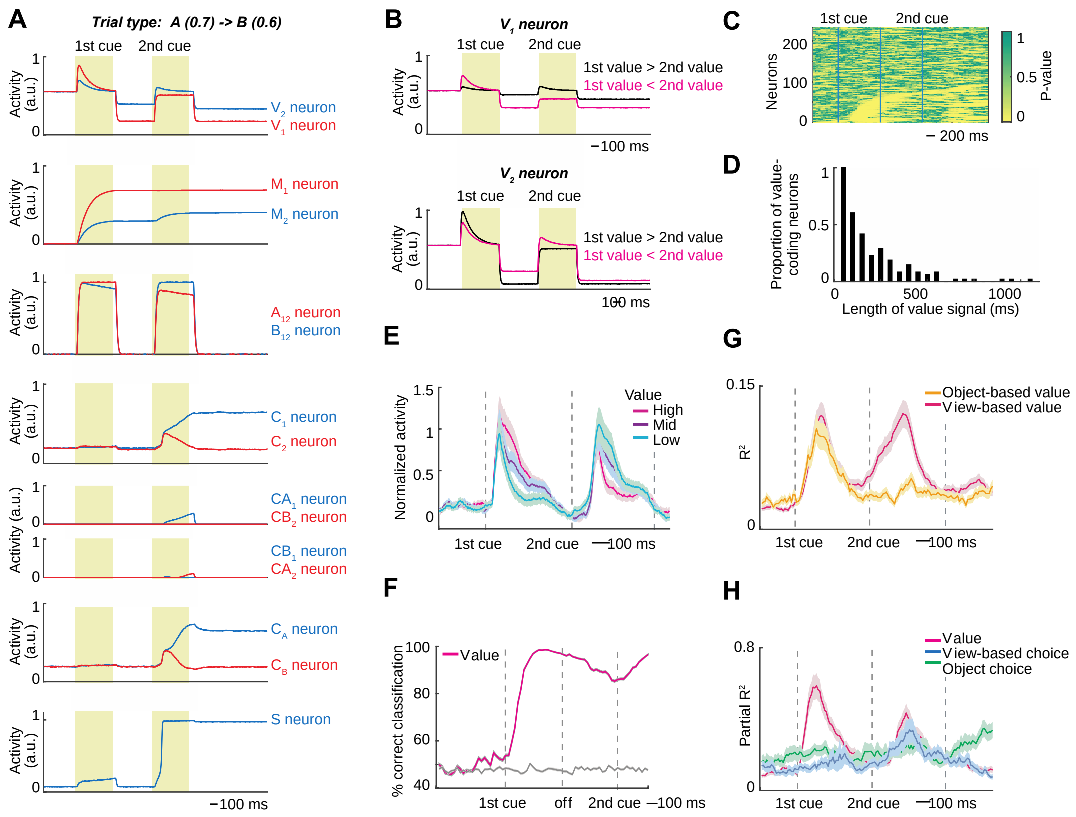

Fig. S10. Overview of model-derived signals and related neuronal data. Related to Fig. 4. **(A)** Model-derived signals for all functional neuron types shown in Fig. 4B. The signals were produced by simulating the model with the object sequence A-then-B, with object values of 0.7 and 0.6 for objects A and B, respectively. **(B)** View-based neurons V_1 and V_2 each signal one input for downstream decision-making per trial. The V_1 neuron has negative value tuning and accordingly responds more strongly to a weaker value input (red), which induces a strong suppression of baseline activity in the delay period via the inhibitory feedback from M_1 memory neurons. The V_2 neuron has positive value tuning and accordingly responds less strongly to a weaker value input (red). This response induces a weaker suppression of baseline activity via the inhibitory feedback from M_2 memory neurons. During the second cue period, the sustained M-to-V inhibition allows the positively tuned V_2 neurons to respond only if the value input by the second object overcomes the inhibition proportional to the first object's value (red); conversely, the negatively tuned V_1 neurons respond only when the second value input is smaller than the first (black). This gating of the second-object response by sustained inhibition ensures that each pool of view-based neurons signals one of the two temporally dissociated values to downstream decision neurons. **(C)** Data from recorded amygdala neurons. P-values for linear regression of single-neuron activity on the value of the first-viewed object, obtained from sliding window analysis (Eq. 7; window size: 200 ms, step size: 20 ms) aligned to the first cue. Data in each row are from a single neuron, sorted from bottom to top within each panel according to coding latency. Across amygdala neurons, value signals varied in latency and duration. Accordingly, value signals in the population bridged the delay period between first and second choice cue. **(D)** Histogram of duration of value signals, defined as the number of consecutive significant sliding windows, calculated from data shown in (C). **(E)** Peri-stimulus time histogram of population activity of 103 neurons coding view-based value (selected based on Eq. 7), sorted according to the value of the first-viewed option. **(F)** Decoding accuracy for view-based value across 233 amygdala neurons during the first stimulus period and in the delay period between first and second stimulus. **(G)** R^2 for 48 neurons coding object value in the first cue period and R2 for 69 neurons coding view-based value in the cue period (selected based on Eq. 6). **(H)** Partial R^2 for view-based value coding neurons (N = 103), view-based choice coding neurons (N = 60), and object-choice coding neurons (N = 75; selected based on Eq. 7).

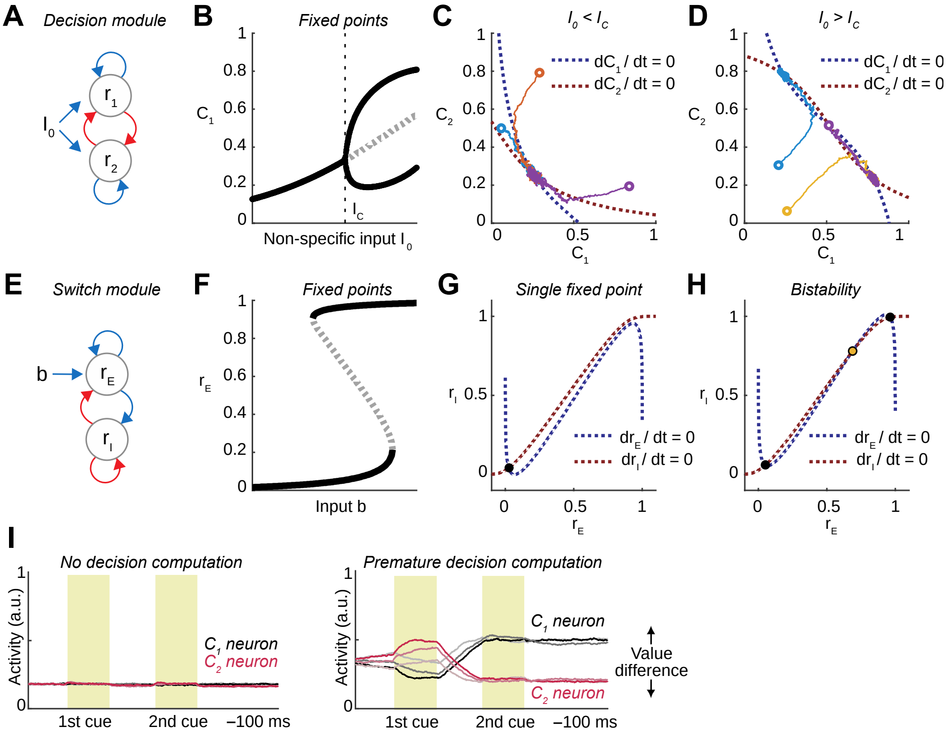

Fig. S11. Model dynamics. Related to Fig. 4. **(A)** The view-based decision subnetwork is composed of two excitatory neural populations that effectively self-excite and mutually inhibit. **(B)** Fixed points of the decision subnetwork. The value of one of the neural populations is indicated as a function of the applied input (*Io*). Due to symmetry the curve is the same for the other neural population. For inputs lower than a critical value I_c, the system settles into a state with equal activity for both populations (no competition). For inputs higher than I_c, the state of no-competition becomes unstable and one of the populations sets into a high-activity state, while the other sets into the low-activity state (competition). Random fluctuations or a biased input favoring one population determine which population wins the competition. Solid lines represent stable fixed points; the dashed line represents unstable solutions. **(C)** For low input ($I_0 < I_c$), competition is absent, and any initial configuration (colored points) will evolve towards a state of equal activity. Fixed points are given by the intersections of the system's nullclines, which are given by $dC_1/dt = 0$ and $dC_2/dt = 0$, respectively. Colored lines represent trajectories in the presence of noise. **(D)** For strong input ($I_0 > I_c$), competition is turned on and the initial configuration determines which population wins the competition. **(E)** The switch subnetwork is composed of interconnected excitatory (E) and inhibitory (I) neural populations. **(F)** Fixed points of the switch subnetwork. The value of the E population is indicated as a function of the applied input (*b*). The system has a low-activity branch and a high-activity branch which coexist in a region of bistability. Thus, an increase in *b* can make the system transit from the state of low activity to a state of high activity. Solid lines represent stable fixed points; the dashed line represents unstable solutions. **(G)** Fixed points are given by the intersections of the system's nullclines, which are given by drE/dt = 0 and drI/dt = 0, respectively. In the low-activity branch and out of the bistability region, the system has a single stable point (black dot). **(H)** In the bistability region, the system has two stable points (black dot) and one unstable point (yellow dot). **(I)** Activity of view-based decision neurons C_1 and C_2 without operation of the switch module. Depending on the background input parameter I_1view-based decision neurons, the model without switch module would either engage in continual, premature decision-making when the value input of the first option was applied, without settling into a stable state (right), or it would not engage in any decision-making due the lack of excitatory drive (left).

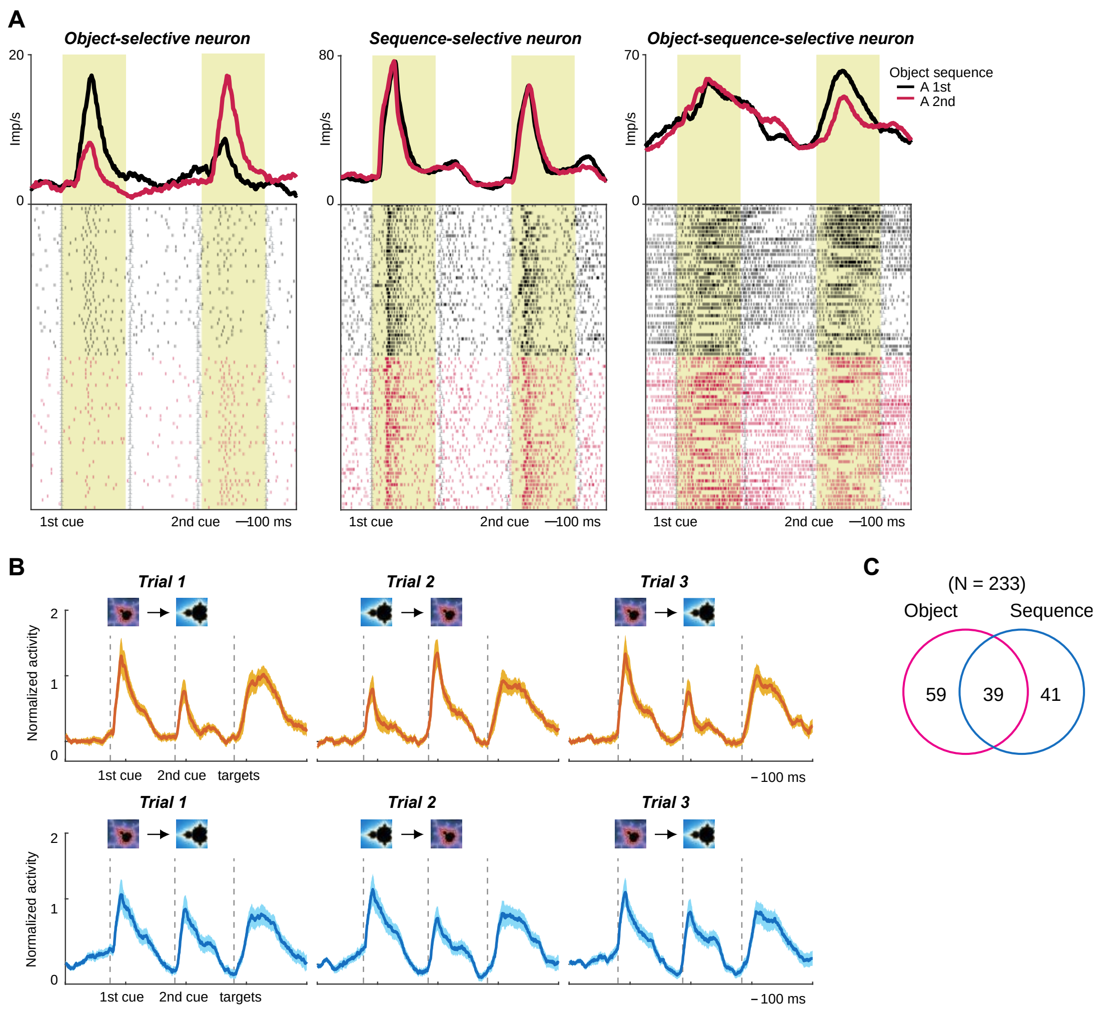

Fig. S12. Amygdala neurons combined information about object identity and viewing sequence. Related to Fig. 4. **(A)** Amygdala neurons with activity related to specific objects (left), the position of objects in a viewing sequence (middle), and the order of a specific object in a viewing sequence (right). **(B)** Population activity of amygdala neurons with activity related to specific objects (top) and sequence position (bottom). Activity (mean ± s.e.m.) was averaged over consecutive trials with varying object sequences. Neurons were identified by regressing each neuron's activity during the sequential object-viewing periods (collapsing across the first and second period) on object identity (object A or object B, coded as 1 and -1), position in viewing sequence (first or second, coded as 1 and -1), and the object-by-sequence interaction (the product of the first two regressors). **(C)** Venn diagram indicating neuron numbers encoding object identity, sequence position, and sequence position for specific objects, identified by the multiple regression model described above.

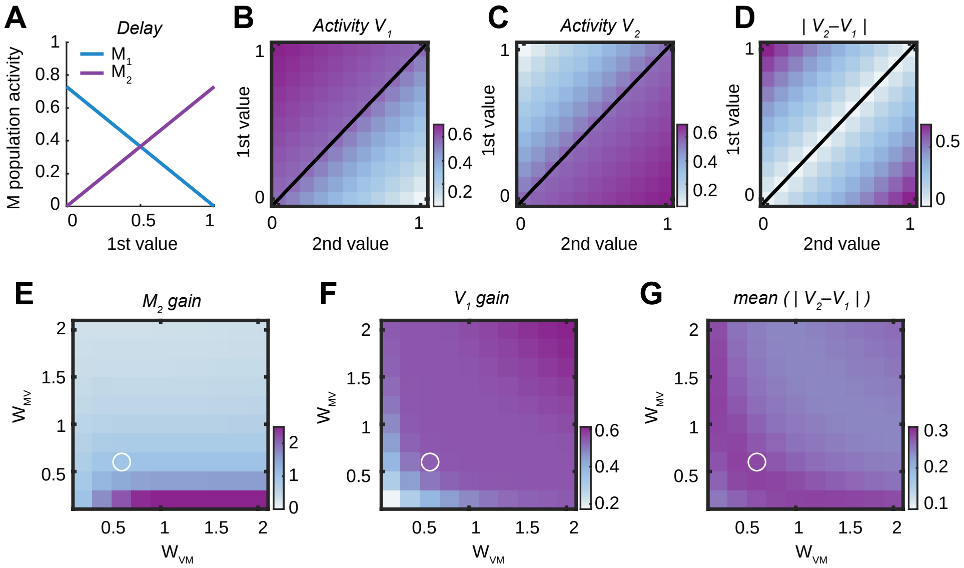

Fig. S13. Dependence of model population activity on value inputs and connectivity parameters. Related to Fig. 4. **(A)** Average activity of neural populations M_1 and M_2 during the delay as a function of the value of the first stimulus. **(B)** Average activity of population V_1 during the presentation of the second stimulus, as a function of the values of the first and the second stimuli. Population V_1 activates when the first value is higher than the second value. **(C)** Same as (B) but for population V_2. Population V_2 activates when the first value is lower than the second value. **(D)** Absolute difference between the activity of V_2 and V_1 during the presentation of the second stimulus, as a function of the values of the first and the second stimuli. $|V_2\text{-}V_1|$ increases as a function of task difficulty. **(E)** The gain of population M2 is defined as the slope of the linear relation between the activity of M2 during the delay and the value of the first stimulus. This gain remains relatively stable (~1) for different choices of connectivity between populations of type V and M (w_{MV} and w_{VM}), but, for low w_{MV}, it increases with increasing w_{VM}. **(F)** The gain of population V1 is defined as the slope of the linear relation between the activity of V1 in response to the first stimulus. This gain depends on w_{MV} and w_{VM}, it increases (decreases) when both w_{MV} and w_{VM} increase (decrease). **(G)** Average absolute difference between the activity of V_2 and V_1 during the presentation of the second stimulus, as a function of w_{MV} and w_{VM}. This measure quantifies the average discriminability between the values of the first and the second stimuli based on the activity of neural populations V_1 and V_2. It remains relatively constant for different choices of w_{MV} and w_{VM}. In (A)–(G), the self-coupling w_{MM} was fixed such that M populations are perfect integrators; the white circle indicates the parameters used in panels (A)–(D) and throughout the study.

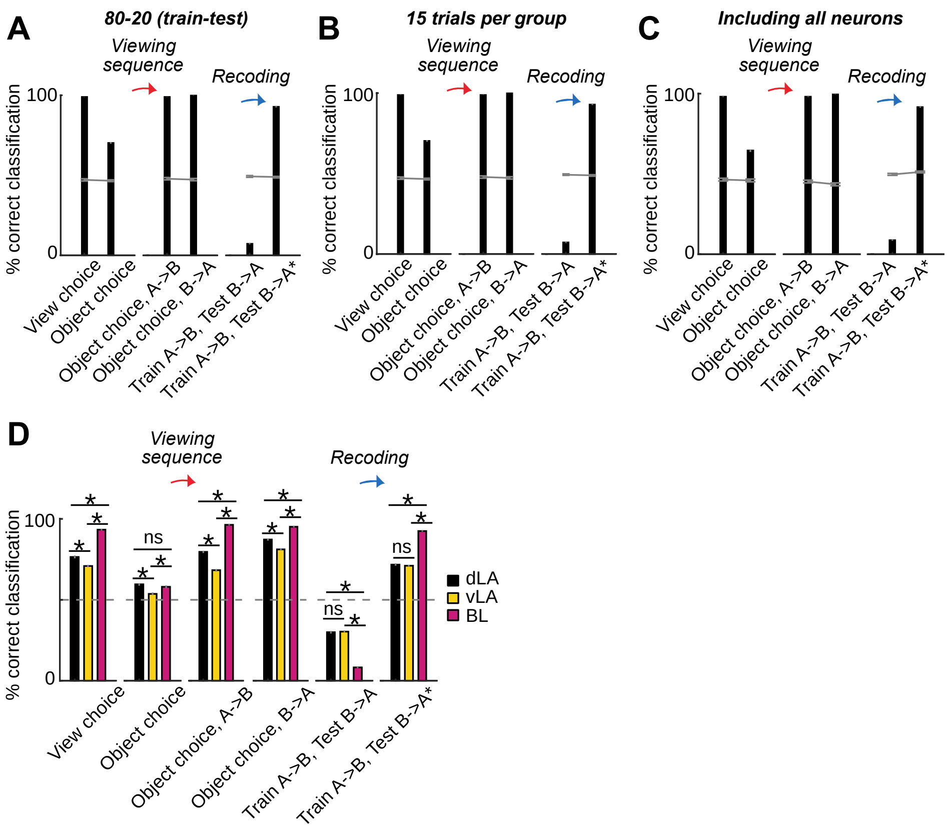

Fig. S14. Decoding robustness tests and comparison of decoding accuracies for view-based choice across amygdala nuclei. Related to Fig. 5. **(A, B, C)** Robustness tests. Results from Fig. 5C were replicated when we varied the parameters of the decoding method (i.e., using 80% of data for decoder training and 20% for decoder testing **(A)**, increasing the inclusion criterion to 15 trials per decoding group **(B),** using all neurons for decoding without pre-selection **(C)**. **(D)** View-based amygdala neurons enable precise decoding of view-based choice but not object choice, particularly in BL (left two panels). Object choice can be decoded when separating the data for specific object-viewing sequences (middle two panels), but not across viewing sequences (second panel from right, 'Train A->B, Test B->A'). Recoding of classification input, by swapping object-classification labels ('Train A->B, Test B->A*'), enables object-choice decoding across viewing sequences (rightmost panel). These effects were strongest in BL. (*: $P < 0.005$, Wilcoxon test).

Supplementary Table S1. Reinforcement-learning models. Related to Fig. 1.

RL model #	% correct	AIC	BIC	Pseudo R^2
Animal A				
1	82.96	125.60	142.86	0.462
2	84.43	113.01	130.71	0.517
3	83.28	123.05	144.49	0.461
4	84.73	113.55	134.52	0.519
5	81.90	130.14	161.61	0.423
6	84.50	120.98	141.85	0.501
7	82.57	130.77	154.82	0.420
8	80.49	131.22	143.23	0.393
**9**	**83.45**	**112.27**	**124.82**	**0.509**
Animal B				
1	80.61	121.95	140.41	0.410
2	82.84	110.08	127.29	0.455
3	81.42	121.65	142.77	0.403
4	82.99	111.72	131.79	0.457
5	80.00	128.77	150.42	0.361
6	83.03	113.46	134.35	0.457
7	78.09	129.28	152.38	0.364
8	77.85	127.92	138.68	0.316
**9**	**81.82**	**104.23**	**116.30**	**0.443**

1: Basic RL; 2: Reversal RL; 3: Basic RL, two learning rates; 4: Reversal RL, two learning rates; 5: Pearce-Hall; 6: Pearce-Hall, reversal learning; 7: Pearce-Hall, two learning rates; 8: Basic RL with reward magnitudes; 9: Reversal RL with reward magnitudes. Numbers in bold indicate the best fitting model.

Supplementary Table S2. Mixed-effects logistic regressions on choice data. Related to Fig. 1.

Variable	Estimate	Standard error	t-statistic	Degrees of Freedom	P-value
Animal A, Eq. 3 (fit to aggregated data)					
Intercept	-0.125	0.047	-2.646	17,081	0.008
1st left	0.141	0.086	1.634	17,081	0.102
1st Mag	0.667	0.031	21.109	17,081	1.1e-97
2nd Mag	-0.586	0.027	-21.081	17,081	2.0e-97
1st Prob	1.302	0.159	8.155	17,081	3.7e-16
2nd Prob	-1.241	0.150	-8.253	17,081	1.6e-16
Animal B, Eq. 3 (fit to aggregated data)					
Intercept	-0.677	0.079	-8.48	5,026	2.9e-17
1st left	0.899	0.157	5.707	5,026	1.2e-8
1st Mag	0.672	0.049	13.569	5,026	3.2e-41
2nd Mag	-0.720	0.062	-11.522	5,026	2.4e-30
1st Prob	1.381	0.282	4.887	5,026	1.0e-6
2nd Prob	-0.948	0.274	-3.451	5,026	5.0e-4
Animal A, Eq. 4 (fit to individual sessions)					
Intercept	-0.031	0.054	-0.585	107	0.559
ObjectAfirst	-0.084	0.029	-2.867	107	0.005
ObjectA RM – ObjectB RM	0.932	0.039	23.862	107	<1.0e-20
ObjectAProb - ObjectBProb	2.546	0.070	36.134	107	<1.0e-20
Animal B, Eq. 4 (fit to individual sessions)					
Intercept	-0.037	0.078	-0.479	35	0.634
ObjectAfirst	-0.199	0.061	-3.294	35	0.002
ObjectA RM – ObjectB RM	1.049	0.073	14.243	35	<1.0e-20
ObjectAProb - ObjectBProb	2.355	0.144	16.254	35	<1.0e-20
